# The Effect of Cardiogenic Factors on Cardiac Mesenchymal Cell Anti-Fibrogenic Paracrine Signaling and Therapeutic Performance

**DOI:** 10.7150/thno.41000

**Published:** 2020-01-01

**Authors:** Justin S. Heidel, Annalara G. Fischer, Xian-Liang Tang, Ghazal Sadri, Wen-Jian Wu, Claudiu R. Moisa, Heather Stowers, Neel Sandella, Marcin Wysoczynski, Shizuka Uchida, Joseph B. Moore IV

**Affiliations:** 1 Diabetes and Obesity Center, Department of Medicine, University of Louisville, Louisville, KY, USA.; 2 The Christina Lee Brown Envirome Institute, Department of Medicine, University of Louisville, Louisville, KY, USA.; 3 Institute of Molecular Cardiology, Department of Medicine, University of Louisville, Louisville, KY, USA.; 4 Cardiovascular Innovation Institute, Department of Medicine, University of Louisville, Louisville, KY, USA.

**Keywords:** Cardiac Mesenchymal Cells, Therapy, Myocardial Infarction, Fibrosis, Paracrine Signaling

## Abstract

Intrinsic cardiogenic factor expression, a proxy for cardiomyogenic lineage commitment, may be an important determinant of donor cell cardiac reparative capacity in cell therapy applications; however, whether and how this contributes to their salutary effects remain largely ambiguous.

**Methods**: The current study examined the consequences of enhanced cardiogenic factor expression, via lentiviral delivery of GMT (GATA4, MEF2C, and TBX5), on cardiac mesenchymal cell (CMC) anti-fibrogenic paracrine signaling dynamics, *in vitro*, and cardiac reparative capacity, *in vivo*. Proteome cytokine array analyses and *in vitro* cardiac fibroblast activation assays were performed using conditioned medium derived from either GMT- or GFP control-transduced CMCs, to respectively assess cardiotrophic factor secretion and anti-fibrogenic paracrine signaling aptitude.

**Results**: Relative to GFP controls, GMT CMCs exhibited enhanced secretion of cytokines implicated to function in pathways associated with matrix remodeling and collagen catabolism, and more ably impeded activated cardiac fibroblast Col1A1 synthesis *in vitro*. Following their delivery in a rat model of chronic ischemic cardiomyopathy, conventional echocardiography was unable to detect a therapeutic advantage with either CMC population; however, hemodynamic analyses identified a modest, yet calculable supplemental benefit in surrogate measures of global left ventricular contractility with GMT CMCs relative to GFP controls. This phenomenon was neither associated with a decrease in infarct size nor an increase in viable myocardium, but with only a marginal decrease in regional myocardial collagen deposition.

**Conclusion**: Overall, these results suggest that CMC cardiomyogenic lineage commitment biases cardiac repair and, further, that enhanced anti-fibrogenic paracrine signaling potency may underlie, in part, their improved therapeutic utility.

## Introduction

Although the cardiovascular medicine field has made great strides in terms of patient care, risk factor identification, and general public health awareness, heart failure (HF) unfortunately remains a major health crisis in the United States and much of the developed world 1. Therapeutic strategies, such as beta-blockers, angiotensin-converting-enzyme (ACE) inhibitors, and aldosterone antagonists continue to be the mainstay treatment for HF patients. Said therapeutic approaches have confirmed clinical utility in alleviating HF symptoms and retarding disease progression; however, they fail to directly target the central etiological mechanisms of HF—cardiomyocyte loss and advancing myocardial fibrosis 2. Thus, there is a grave need for the development of innovative and effective therapeutic strategies that directly hinder disease development and promote cardiac functional recovery in HF patients. Cardiac cell-based therapies were introduced in effort to address the deficiencies of traditional pharmacological approaches. And since its inception more than a decade ago, a number of studies have reported a calculable benefit with cell therapy on detrimental ventricular remodeling and functional recovery in preclinical models of heart failure 3-9. But while these studies indicate a tangible and reproducible improvement in ventricular performance after cell administration, their fundamental underlying mechanisms of action, as well as the essential characteristics that contribute to their reparative capacity, remain elusive. The cell therapy field has become saturated with an ever-growing number of supposed cardiomyogenic stem or progenitor cells, which could in theory be leveraged to replenish cardiomyocyte populations lost after cardiac ischemic injury. However, there is limited reliable evidence that any of these “cardiac progenitor cells,” even those well established to competently generate cardiomyocytes *in vitro* (i.e., embryonic stem cells or induced pluripotent stem cells), are sufficiently retained and/or meaningfully contribute to the remuscularization of the post-infarcted mammalian heart after administration 10-14—indicating a plausible paracrine-mediated mechanism of action. Irrespective of these reports, many of these cell populations with cardiac potential or rather those oriented towards a cardiovascular phenotype, including c-kit+ enriched 12, 13, 15 or unfractionated cardiac mesenchymal stromal cells (CMCs) 16-20, have been verified as therapeutic agents that improve cardiac function and assuage detrimental remodeling. It is, thus, not beyond reason to postulate that cells with cardiac potential—without ever taking on the form of an adult myocyte—could favorably contribute to cardiac repair. What is more, it is also plausible that this cardiac potential is an important determinant of a cell's cardiac reparative capacity.

There is in fact some credence to the idea that cardiogenic potential, or rather cardiomyogenic lineage commitment, is an important determinant of donor cell reparative capacity. For instance, previous investigations implicate the expression of core cardiogenic transcription factors (e.g., Nkx2.5, Tbx5, Mef2c, etc.) in donor cells to favor cardiac repair 18, 20, 21. And while the precise mechanisms by which the expression of these factors contribute to the therapeutic actions of donor cell populations remain unknown, said findings lend credibility to the idea that enhancing myogenic lineage commitment could be an effective means to boost donor cell therapeutic utility. This philosophy has been the keystone of multiple studies assessing the proficiency of various techniques to “forward reprogram” or coax mesenchymal progenitors toward a cardiomyocyte-like fate. Such methods have included ectopic expression of cardiogenic transcription factors (GMT: Gata4, Mef2c, and Tbx5) 22, 23, as well as exposure to chromatin modifying agents—like histone deacetylase (HDAC) 24, 25 and DNA methyltransferase (DNMT) 26-28 inhibitors. Following this course of investigation, we previously identified HDAC1 as an important mediator of CMC cardiovascular lineage specification 25 and paracrine signaling potency 29, findings which have implicated HDAC1 as a potential therapeutically exploitable target to boost the cardiac reparative aptitude of CMCs. Such inferences have prompted a recent follow-up study examining the effects of pharmacologic HDAC1 inhibition on CMC lineage specification and therapeutic efficacy *in vivo* 20. There, we discovered that CMCs pre-treated with the isoform-selective HDAC1 inhibitor, entinostat, possessed elevated expression of core cardiogenic transcription factors, heightened capacity for myocyte-like differentiation *in vitro*, and improved ability to attenuate systolic dysfunction in a rat model of chronic ischemic cardiomyopathy 20. Enhancement in CMC reparative capacity with entinostat exposure was not associated with improved cell retention, cardiomyogenesis, and/or angiogenesis, but rather with greater inhibition of ventricular fibrosis. Congruent with these observations, entinostat-treated CMCs exhibited augmented secretion of a host of soluble factors implicated in matrix remodeling and collagen catabolism—suggesting that their improved actions *in vivo* may, in part, be associated with lineage commitment-mediated adaptations in CMC anti-fibrogenic paracrine signaling. Though pharmacologic HDAC inhibition afforded a measurable boost in CMC cardiac reparative capacity, whether this phenomenon was the direct result of enhancement in CMC cardiomyogenic lineage commitment could not be ascertained from this study alone, as there are a multitude of other known and unknown substrates subject to HDAC regulation. To this end, in the current study, we sought to employ a more direct approach to understand whether, and to what extent, cardiomyogenic lineage commitment influences CMC paracrine signaling dynamics and therapeutic performance. More specifically, the scope of the current study was to examine the effects of enhanced cardiomyogenic lineage commitment, by means of ectopic expression of GMT cardiogenic transcription factors, on CMC cardiotrophic factor secretion and anti-fibrogenic paracrine signaling potency—and whether such alterations in paracrine signaling dynamics translate to improved cardiac reparative capacity *in vivo*.

## Methods

### Ethical conduct of study

All animal experiments performed herein were approved and carried out in strict compliance with the Guide for the Care and Use of Laboratory Animals published by the U.S. National Institutes of Health (Eight Edition, Revised 2010), and with the guidelines of the Animal Care and Use Committee of the University of Louisville, School of Medicine (Louisville, KY, USA). Stated committee approvals and the methods by which these experiments were performed were in direct compliance with the ethical standards laid down in the 1964 Declaration of Helsinki and its later amendments.

### Rat Cardiac Mesenchymal Stromal Cell/Fibroblast Isolation

Fischer-344 (F-344) rat CMCs were isolated from whole heart specimens according to a previously established collagenase II digestion procedure 25, 29. Briefly, whole hearts were surgical resected from isoflurane inhalant anesthetized F-344 rats, transferred to sterile 10 cm dishes, and mechanically minced using Metzenbaum scissors. Minced myocardial tissue was subsequently washed via gravity sedimentation in 1X PBS solution to remove red blood cells. Minced myocardial tissue was then enzymatically digested in 10 mL of collagenase II (7 mg/mL in 1X PBS; Worthington Labs), with gentle agitation for 45 min at 37 ⁰C. Enzymatic digestion was halted via the addition of 40 mL of inactivation medium (DMEM/F12, 10% FBS). Resultant cells liberated from digested tissues were harvested by centrifugation at 600 g for 10 min and then washed in 1X PBS. Lastly, cell pellets were gently resuspended in CMC complete medium [Ham's F12 medium (Gibco) supplemented with 10% FBS (VWR Life Science Seradigm Fetal Bovine Serum), 20 ng/mL recombinant human bFGF (PeproTech), 1X GlutaMAX™ (Gibco), and 100 U/mL penicillin/streptomycin (Gibco)], transferred to T75 tissue grade culture flasks, and placed in a cell culture incubator for expansion (passage 0).

### Cells, Cell Culture, and Passaging

Rat CMCs were cultured in CMC complete medium (as described above). All cell lines were maintained under standard incubation conditions at 37 ⁰C with 5% atmospheric CO_2_ and passaged using TrypLE™ (Thermo Fisher Scientific) when approaching ≈70-80% confluency. Rat CMCs were not used in experiments beyond passage number 8.

### Tricistronic Cardiac Transcription Factor Lentiviral Vector Construction

GATA4, MEF2C, and TBX5 cardiac transcription factor coding sequences, as employed previously 23, were assimilated into a tricistronic lentiviral expression vector. These included verified full-length cDNA clones: human MEF2C cDNA [MHS1010-7295133; Clone ID: 4815933; NCBI Accession: BC026341)] (Open Biosystems), human TBX5 cDNA [MHS1010-7430001; Clone ID: 5204163; NCBI Accession: BC027942] (Open Biosystems), and murine GATA4 cDNA [NCBI Accession: NM_008092.3] (sourced from the Dr. Deepak Srivastava laboratory; University of California, San Francisco). Coding sequences were PCR-amplified (Phusion® High-Fidelity PCR Kit; New England BioLabs, Inc.) from previously established 23 pLenti6/V5-D-TOPO vectors (Invitrogen) individually harboring GATA4, MEF2C, or TBX5 cDNA. A puromycin resistance cassette was similarly amplified from a pLK0.1-Puro vector (Sigma-Aldrich). Resultant PCR amplicons (GATA4, MEF2C, TBX5, and hPGK-PuroR) were sequentially restriction cloned into the pUltra 3^rd^ generation lentiviral vector—kindly provided by Dr. Malcolm Moore (Addgene plasmid #24129; http://n2t.net/addgene:24129; RRID: Addgene_24129) 30. Cloning primers, primer-specific restriction enzymes, and template DNA used in amplification procedures are detailed in Supplemental Table S1.

### Lentiviral Production and CMC Transduction

pUltra-GMT or pUltra-GFP control lentiviral particles were derived using the ViraPower™ Lentiviral Expression System (Life Technologies) according to the manufacturer's instructions. Approximately 2x10^5^ rat CMCs were plated in 10 cm tissue culture grade dishes using Ham's F12 complete medium prior to cell transduction. At 24 h (day 0), 9 mL of diluted viral supernatant (3 mL viral supernatant [MOI, 1.6-2.3; viral titer 270,000-380,000 TU/mL] in 6 mL Ham's F12 complete medium) containing 8 µg/mL of polybrene was added to CMCs and incubated for an additional 24 h (post viral transduction day 1). Viral medium was then replaced with Ham's F12 complete medium and incubated for an addition 48 h. On post viral transduction (day 3), complete medium was replaced with antibiotic selection medium (Ham's F12 complete medium supplemented with 1 µg/mL puromycin). Virally transduced CMCs (GFP CMCs and GMT CMCs) were maintained under 1 µg/mL puromycin antibiotic selection throughout their duration in culture. Viral transduction efficiency and the fidelity of puromycin selection were routinely evaluated via fluorescent-mediated detection of green fluorescent protein (GFP) in CMCs transduced with pUltra-GFP control. GFP and GMT CMCs were maintained under standard incubation conditions for 2 weeks prior to being harvested for intramyocardial injection procedures.

### Western Blotting

Immunoblotting was performed according to previously described protocols 25, 29. A detailed list of antibodies with corresponding dilutions is available in Supplemental Table S2.

### Quantitative PCR

Total RNA was isolated and prepared using a PureLink^®^ RNA Mini Kit (Life Technologies) per the manufacturer's instructions. Total RNA was reverse transcribed using the SuperScript^®^ III First-Strand Synthesis System (Life Technologies), according to the manufacturer's protocol with random hexamer primers. Quantitative PCR was performed with Excella^®^ SYBR^®^ MasterMix, Rox^™^ in a QuantStudio5 real-time PCR system (Applied Biosystems). Gene specific primer sets used for the detection of cardiac markers include: α-MHC (forward: 5´-GACATCCGCACAGAGTGCT-3´ and reverse: 5´-CCTGGTCCTCCTTCACAGTC-3´); β-MHC (forward: 5´-AGAAGATGGTGTCCCTGCTG-3´ and reverse: 5´-CCAGCTGGATCTTGTTCTTGA-3´); β-actin reference (forward: 5´-CCATCATGAAGTGTGACGTTG-3´ and reverse: 5´-AGGAGCCAGGGCAGTAATCT-3´). PCR was performed with an initial denaturation step of 95 ^o^C for 10 min, followed by 40 cycles of 15 s denaturation at 95 ^o^C and 1 min annealing/extension at 60 ^o^C. Fold expression was calculated according to the ΔΔCT method for quantitative real-time PCR using β-actin as an internal reference control.

### CMC Conditioned Medium Harvest and Cytokine Array Analyses

CMC condition medium was harvested according to the previous established protocol 20. In brief, 1 x 10^6^ CMCs were seeded in T75 tissue culture flasks with complete medium. When cultures reached approximately 70-80% confluency, cells were rinsed with 1X PBS and then incubated with serum-free, Ham's F12 medium (Gibco) supplemented with 0.5% bovine serum albumin (BSA) for 24 h. Conditioned medium was harvested via a sterile serological pipette and transferred to 15 mL conical tubes. Detached cells were removed via 600 g centrifugation for 10 min at 4 ⁰C. Cytokine arrays were performed using the Proteome Profiler™ Rat XL Cytokine Array Kit (Cat. # ARY030, R&D Systems, Minneapolis, MN), per the manufacturer's protocol. For each experimental replicate, a total of 1 mL of unconcentrated cell culture supernatant/conditioned medium was used. Resultant membranes were imaged with a chemiluminescent image analyzer (MyECL; Thermo Fisher Scientific) and analyzed by densitometric quantification (ImageJ; NIH, Bethesda, MD). Pixel density values were imported into R version 2.15.1 x 64 bit (The R Project for Statistical Computing) and subject to hierarchical cluster analysis using the heatmap.2 function in the gplots library. Each conditioned medium experiment (corresponding to either GMT-transduced or GFP-transduced CMCs) was performed using independent biological replicates (n=2 for each). Cytokines identified to be more abundant in conditioned medium sourced from GMT CMCs (compared to GFP-transduced controls) were subject to functional annotation clustering using the Database for Annotation, Visualization and Integrated Discovery (DAVID) 31, 32.

### TGFβ1-mediated Cardiac Fibroblast Activation Assays

F-344 rat cardiac fibroblasts (passage ≤ 3) were seeded in 6-well tissue culture plates at a number of 2.5 x 10^5^ cells per well using CMC complete medium. After 24 h, cardiac fibroblasts were serum starved for 2 h by replacing culture medium with 1 mL of serum-free, Ham's F12 basal medium (Gibco). Next, cardiac fibroblasts were incubated with 2 mL of GMT or GMT CMC conditioned medium supplemented with fibroblast activating peptide, TGFβ1 (20 ng/mL; Gibco; Cat. PHG9204). Concurrently, cardiac fibroblasts incubated with basal Ham's F12 medium supplemented with recombinant human TGFβ1 (20 ng/mL; Gibco; Cat. PHG9204) or recombinant human bFGF (20 ng/mL; PeproTech, Inc.; Cat. 100-18B) served as positive and negative cardiac fibroblast activation controls, respectively. After 48 h, cardiac fibroblasts were harvested, total protein extracts collected, and the expression of fibroblasts activation markers (e.g., α-SMA, Col1A1, and Col19A1) evaluated via immunoblotting. A detailed list of employed antibodies with corresponding dilutions is available in Supplemental Table S2.

### Ischemia Reperfusion (I/R) and CMC Injection Procedures

Similar to our previously performed rat ischemia-reperfusion injury procedure 20, female F-344 rats (supplier, Charles River Laboratories; age, 2-3 months; weight, 150-180 g) were anesthetized with ketamine (37 mg/kg) and xylazine (5 mg/kg) and intubated with a rodent respirator (Harvard Apparatus). Anesthesia was maintained with isoflurane inhalation at a fixed body temperature of 37 ⁰C using a heating pad. After intravenous administration of antibiotics, the chest was opened via left thoracotomy and the heart exposed. All animals were subsequently subject to 120 min occlusion of the left anterior descending coronary artery followed by reperfusion, after which time the chest wall was sutured closed. Thirty days after reperfusion, rats were re-anesthetized, and the heart exposed via left anterolateral thoracotomy. At this interval, rats received intramyocardial (peri-infarct) injections of GFP CMCs, GMT CMCs, or vehicle (1X PBS; phosphate-buffered saline). To limit potential cell line-dependent variables, each rat received a mixture of CMCs (GFP-transduced or GMT-transduced) derived from three independently established rat CMC cell lines. Of these three CMC cell lines, each was individually transduced with independently prepared pUltra-GFP or pUltra-GMT lentiviral particles on different d. CMC-treated animals received a total of 2 million CMCs per heart (6 injections per heart; approximately 333,000 cells per injection). All groups were followed for an additional 35 d after injection and compassionately euthanized (65 d after MI).

### Power Analyses, Exclusion Criteria, and Randomization

Sample size was calculated via statistical power analysis in SigmaStat Software (Systat Software, Inc.). Accordingly, 30 rats (aged 2-3 months) were required for successful completion of the *in vivo* component of the study. Calculations were performed with a requisite power of 80% and a type I error α=0.05. An exclusion criterion, established prior to the study's commencement, dictated any animal lacking a ≥ 15% reduction in left ventricular ejection fraction (ΔEF) 30 d after ischemic injury relative to baseline be withdrawn from the study. Based on this criterion, 31 animals were accepted for the study and correspondingly assigned to the treatment groups (GFP CMC or GMT CMC; n=11 and n=10, respectively) or vehicle control (1X PBS; n=10) at random using the Mersenne Twister algorithm in Microsoft Excel.

### Echocardiographic Procedures and Analyses

Indices of cardiac function and ventricle dimensions were evaluated via echocardiography under light anesthesia (pentobarbital, 25 mg/kg, intraperitoneal, as previously described) 13, 33 with serial measurements acquired at baseline (prior to I/R; Baseline), 30 d post-I/R (prior to treatment; Pre-Tx), and 65 d post-I/R (35 d after treatment; Post-Tx). The anterior chest was shaved, and the rat then placed in the left lateral decubitus position. Body temperature was maintained between 36.9 ⁰C and 37.3 ⁰C. Echocardiographic images were acquired using an HP SONOS 7500 ultrasound system outfitted with a L12-5 linear broadband and an S12 phased array transducer equipped with a 0.3 cm standoff. Hearts were imaged in the para-sternal long axis view to measure LV end-systolic and end-diastolic volumes (LVESV and LVEDV, respectively), as well as ejection fraction (EF). All measurements were averaged in three consecutive cardiac cycles and analyzed off-line by a single blinded observer using COMPACS image analysis software. All calculations were derived using standard formulas and analyzed according to modified American Society for Echocardiography standards 34. Echocardiograms were collected from all 31 animals, which consisted of vehicle control (1X PBS; n=10) and CMC treatment groups (GFP CMCs or GMT CMCs; n=11 and n=10, respectively).

### Hemodynamic Procedures and Analyses

Hemodynamics measurements were performed 65 d after MI, just prior to euthanasia as previous 20. Rats were anesthetized with ketamine (37 mg/kg) and xylazine (5 mg/kg), intubated, and mechanically ventilated. Anesthesia was sustained under 1% isoflurane with core temperatures monitored and preserved at 37 ⁰C throughout the procedure's duration. A 2F Mikro-Tip® ultra-miniature pressure-volume (PV) loop catheter (SPR-869, Millar Instruments) was inserted into the right carotid artery and advanced into the LV cavity. The right jugular vein was cannulated for fluid administration. Following a 20 min stabilization period, PV signals were continuously recorded with an ARIA-1 single-segment PV conductance system (Millar Instruments) interfaced to a Powerlab/4SP data acquisition system (ADinstruments). PV relationships were evaluated via transient compression of the inferior vena cava with a cotton swab. Parallel conductance from surrounding structures was calculated by injecting a small bolus of 15% NaCl through the jugular vein. LV end-diastolic pressure (LVEDP), dP/dt_max_ and dP/dt_min_, end-systolic elastance (Ees), and pre-load adjusted maximal power were calculated using the PVAN software program, as previously 12, 13. Conductance catheterization was attempted on all 31 animals; however, one GMT CMC-treated rat died during surgical procedures, which brought group totals to n=10 for vehicle, n=11 for GFP CMC, and n=9 for GMT treatment groups.

### General Histology and Morphometry Procedures

In accordance with previous histopathology procedures 20, at the conclusion of hemodynamic measurements, a polyethylene catheter filled with phosphate buffer (0.2 M, pH 7.4) and heparin (100 IU/mL) was advanced into the ascending aorta through the right carotid artery. In rapid succession, the heart was arrested in diastole via 1 mL injection of a solution of CdCl_2_ (100 mM) and KCl (3 M) through the aortic catheter. Afterward, the heart was excised, retrogradely perfused with phosphate buffer for 3 min, and perfused with 10% neutral buffered formation for 15 min. Perfusion pressures were maintained between 60 and 80 mmHg while end-diastolic pressures were retained at 8 mmHg. After perfusion-fixation, whole heart mass was measured and recorded. Each heart was then successively transversally dissected into 5 slices (each, 3 mm in thickness), processed, paraffin-embedded, section at 4 µm intervals, and stained with Masson's trichrome or Picrosirius red.

### Infarct Size Determination by Masson's Trichrome Staining

Trichrome staining was performed as directed in the manufacturer's protocol (HT15-1KT; Sigma-Aldrich), as previously performed 20. In brief, formalin-fixed, paraffin embedded myocardial tissue sections were heated for 30 min at 80 ⁰C, deparaffinized in xylene, and stepwise rehydrated via incubation in decreasing concentrations of ethanol (100, 96, 90, 80, and 0%). Tissue sections were subsequently incubated in Richard-Allan Scientific™ Bouin's Fluid (57211, Thermo Fisher Scientific) for 15 min at 55 ⁰C and allowed to cool for 5 min. Myocardial sections were then washed with deionized water for 15 min, allowed to rest in fresh deionized water for 5 min, and stained with Biebrich Scarlet-Acid Fuchsin solution (HT151; Sigma-Aldrich) for 10 min. Tissue sections were subsequently rinsed in deionized water for 5 min and incubated in working phosphotungstic acid (HT152; Sigma-Aldrich)/phosphomolybdic acid (HT153; Sigma-Aldrich)/deionized water solution for 5 min. Myocardial tissue sections were successively counter-stained with aniline blue (HT154; Sigma-Aldrich) for 1 min, rinsed in 1% acetic acid for 1 min, and stepwise dehydrated with incubation in increasing concentrations of ethanol (80, 90, 96, and 100%). After rehydration, tissues sections were mounted under glass coverslips using Permount Mounting Medium (SP15-100; Thermo Fisher Scientific). Images were digitally acquired at 1X magnification using a Nikon Eclipse Ni-E light microscope and analyzed using NIH ImageJ software (1.46r). Morphometric parameters, such as total LV area, remote area, risk area, and scar area, were measured in each section. In accordance with our previous work 12, the risk region was defined as the sum of the LV segment containing the infarct scar and the two border zones (the region that includes 0.5 mm on either side of the lateral borders of the scar). Total scar size (mass in mg) was enumerated by summation of scar masses corresponding to each transverse myocardial slice (apex to base; five slices per heart):


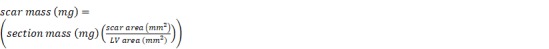


### Quantification of Myocardial Collagen Content by Picrosirius Red Staining

To assess cardiac fibrosis, myocardial collagen content was enumerated in Picrosirius red stained tissue sections via quantitative analysis of polarized light microscopy images, according to the previous protocols 20. Formalin-fixed, paraffin-embedded myocardial tissue sections were heated 80 ⁰C for 30 min, deparaffinized in xylene, and stepwise rehydrated via incubation in decreasing concentrations of ethanol (100, 96, 90, 80, and 0%), and placed in deionized water. Picrosirius red stain was prepared using 0.1% (w/v) Direct Red 80 (365548-5G; Sigma-Aldrich) in picric acid (P6744-1GA; Sigma-Aldrich). For each heart, mid-papillary muscle level myocardial sections were incubated in Picrosirius red for 1 h, washed in 0.5% acetic acid (2 times; 1 min each), stepwise dehydrated with ethanol, and mounted under glass coverslips using Permount Mounting Media (SP15-100, Thermo Fisher Scientific). Images were digitally acquired using a Nikon Eclipse Ni-E microscope at both 1X magnification under brightfield and at 4X magnification under polarized light. Acquired polarized light images (for each myocardial section) were digitally reconstructed using the Stitching Plug-in for NIH ImageJ (1.46r) 35; afterward, a signal threshold was established and applied to all acquired polarized light images (including stained sections from all groups) prior to digital quantification in ImageJ (1.46r). Results are expressed as the percent area of collagen per myocardial tissue region ± SEM.

### Statistical Analyses

Statistical analyses were performed using GraphPad Prism version 7.00 for Windows (GraphPad Software, San Diego California USA, http://www.graphpad.com). All data sets were subject to formal tests for normality using the D'Agostino-Pearson test. Immunoblot densitometric data were analyzed for statistical significance with the one-way ANOVA followed by the post hoc Holm-Sidak's multiple comparisons test. Echocardiographic and hemodynamic data were evaluated by one- or two-way Analysis of Variance (ANOVA), where appropriate, followed by the *post hoc* Holm-Sidak's multiple comparisons test. Infarct size (assessed from Masson's trichrome staining) and myocardial collagen content (from picrosirius red) data were assessed by 1-way ANOVA followed by the *post hoc* Holm-Sidak's and Tukey's multiple comparisons test, respectively. The arithmetic mean ± SEM is reported for all data sets. Calculated probability values of less than 0.05 were considered statistically significant.

## Results

### Tricistronic GMT vector construction and validation

**Figure 1:**
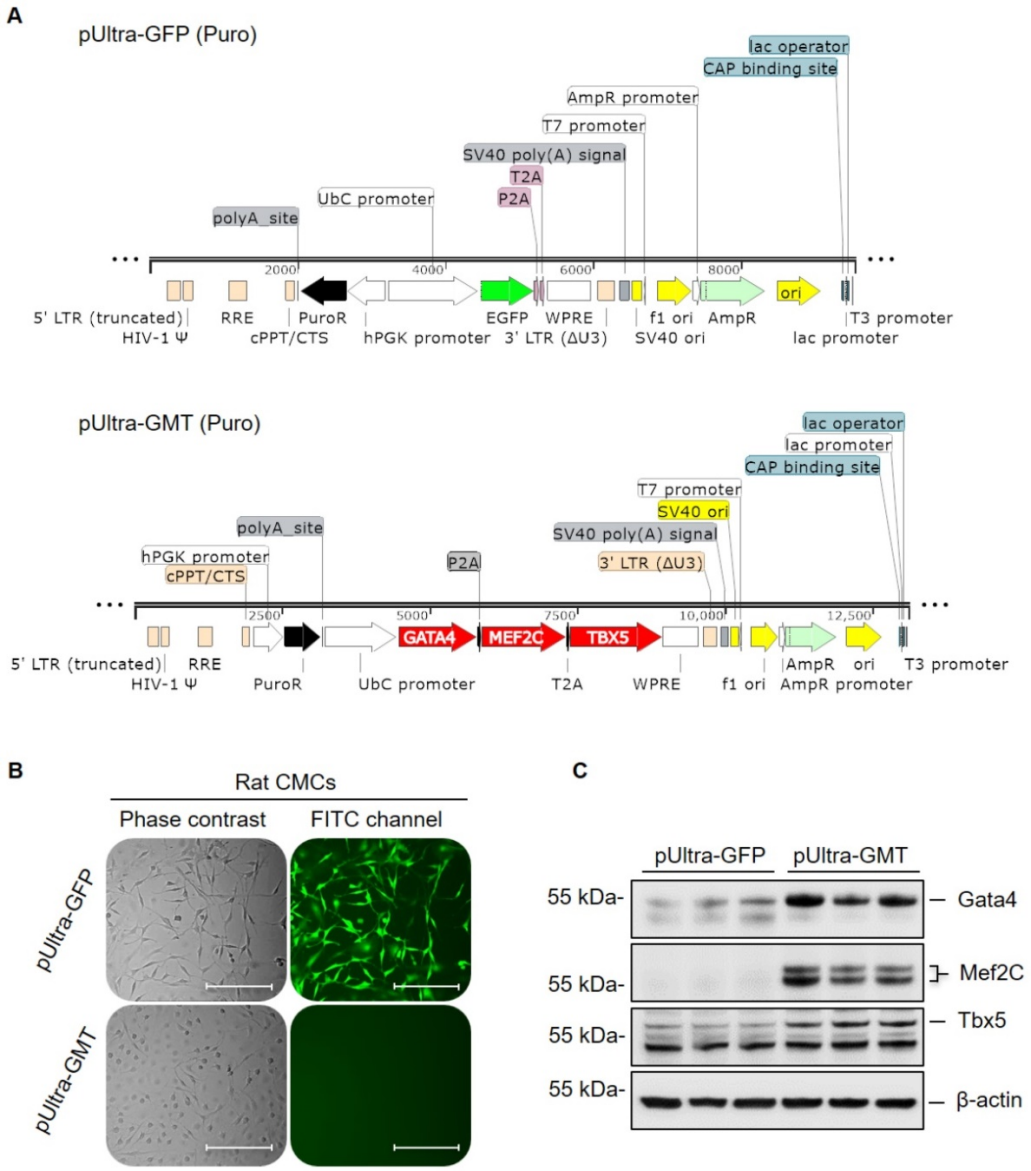
** GMT cardiogenic transcription factor expression vector construction and viral transduction.** (A) pUltra-GFP (top panel) control and pUltra-GMT (bottom panel) tricistronic lentiviral expression vectors (images generated using SnapGene®). (B) Phase contrast and FITC fluorescence microscopy images of pUltra-GFP and pUltra-GMT transduced CMCs. Scale = 200 µm. (C) Representative Gata4, Mef2C, Tbx5, and β-actin (loading control) immunoblots using total protein lysates harvested from lentiviral-transduced rat CMCs (n=3 biological replicates).

Gata4, Mef2c, and Tbx5 (referred to as GMT hereafter) comprise a defined set of cardiogenic transcription factors whose ectopic expression in adult cardiac mesenchyme is sufficient to induce cardiogenic transcriptional program activation and myocyte-like lineage specification 22, 23. To this end, previous experimentally validated GMT coding sequences 23 were sequentially restriction cloned into a polycistronic pUltra lentiviral construct (Figure 1A); said coding sequences were separated by P2A and T2A “self-cleaving” peptides, allowing for successive translation of Gata4, Mef2c, and Tbx5 proteins from a single tricistronic mRNA (Figure 1A). Rat CMCs were subsequently transduced with lentiviral particles harboring either pUltra-GMT or pUltra-GFP (Figure 1A) control constructs and subjected to sustained puromycin selection (Figure 1B). The fidelity of puromycin selection was confirmed via fluorescence imaging of pUltra-GFP-transduced CMCs (Figure 1B). Phase contrast images of GFP CMCs showed conservation of characteristic mesenchymal spindle morphology, whereas GMT CMCs exhibited a more planar appearance with fewer cellular projections (Figure 1B). This phenotype correlated with heightened expression of GMT core cardiogenic transcription factors, as evidenced by their antibody-mediated detection in isolated total protein lysates (Figure 1C). Immunoblots in Figure 1C confirmed efficient and robust ectopic expression of Gata4, Mef2c, and Tbx5 recombinant proteins in GMT CMCs, compared to GFP CMC controls, which exhibited minimal expression of these factors. The functionality of these factors was confirmed via qPCR-mediated detection of cardiac myosins (Figure S1). Consistent with a previous report 36, viral transduction resulted in a statistically significant induction of α-MHC mRNA expression (≈110% increase), but not β-MHC, in GMT-transduced CMCs relative to GFP controls.

### GMT CMCs exhibit augmented anti-fibrogenic paracrine signaling potency *in vitro*

**Figure 2:**
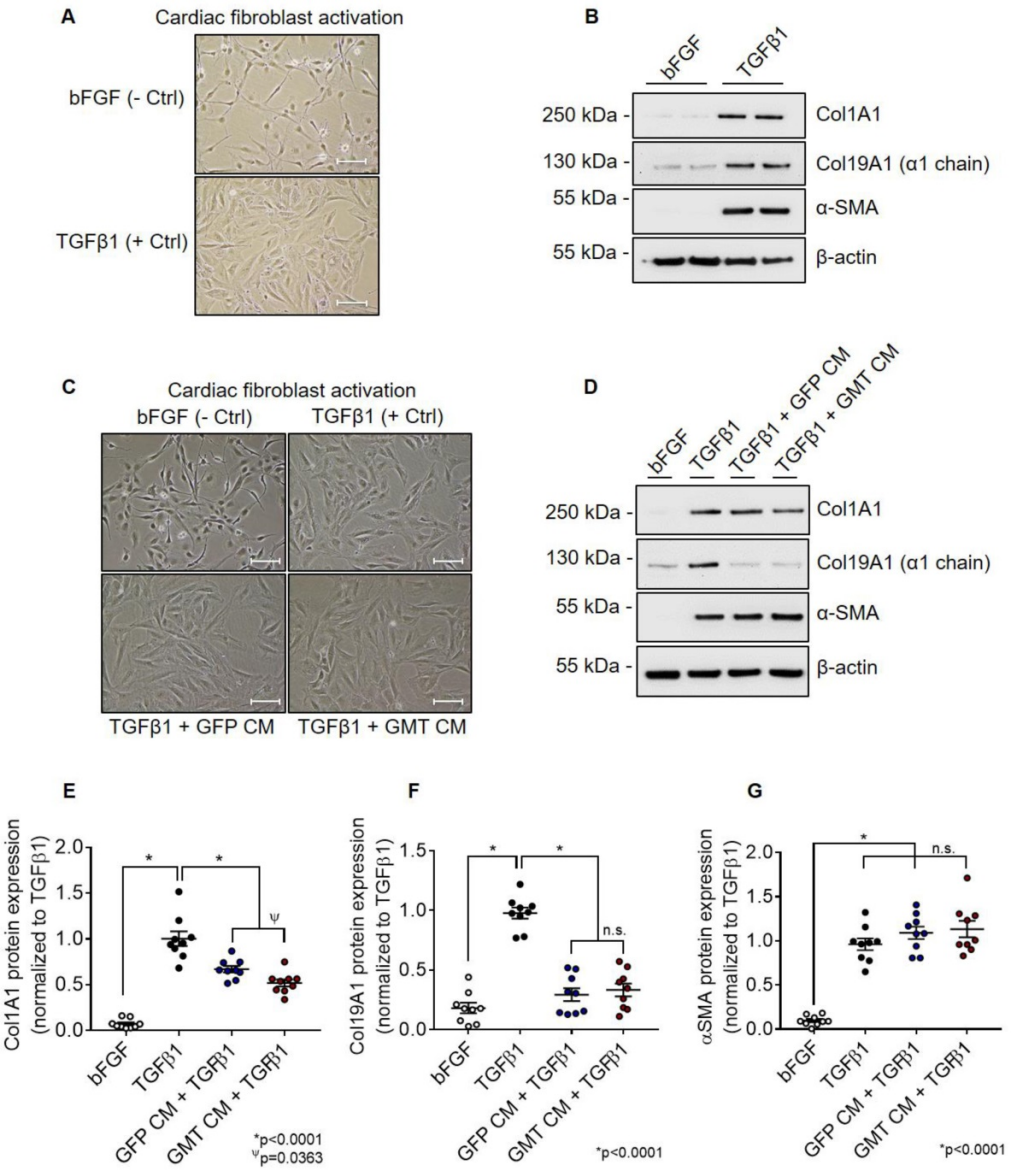
** GMT CMCs display enhanced anti-fibrogenic paracrine signaling potency *in vitro*.** (A) Representative phase contrast microscopy images of activated (TGFβ1; + Ctrl) and unstimulated (bFGF; - Ctrl) rat cardiac fibroblasts. Scale = 100 µm. (B) Representative Col1A1, Col19A1, α-SMA, and β-actin (loading control) immunoblots using total protein lysates harvested from activated (TGFβ1; + Ctrl) and unstimulated (bFGF; - Ctrl) rat cardiac fibroblasts. (C) Representative phase contrast microscopy images of activated cardiac fibroblasts (TGFβ1; + Ctrl), unstimulated cardiac fibroblasts (bFGF; - Ctrl), activated fibroblasts co-incubated with GFP CMC conditioned medium (TGFβ1 + GFP CM), and activated fibroblasts co-incubated with GMT CMC conditioned medium (TGFβ1 + GMT CM). Scale = 100 µm. (D) Representative Col1A1, Col19A1, α-SMA, and β-actin (loading control) immunoblots using total protein lysates harvested from activated cardiac fibroblasts (TGFβ1; + Ctrl), unstimulated cardiac fibroblasts (bFGF; - Ctrl), activated fibroblasts co-incubated with GFP CMC conditioned medium (TGFβ1 + GFP CM), and activated fibroblasts co-incubated with GMT CMC conditioned medium (TGFβ1 + GMT CM). Densitometric quantification of (E) Col1A1, (F) Col19A1, and (G) αSMA immunoblots using total protein extracts derived from bFGF, TGFβ1, GFP CM + TGFβ1, and GMT CM + TGFβ1 rat fibroblast treatment groups. n = 9 biological replicates for each group. All graphs report mean ± SEM. Data were analyzed for statistical significance via 1-way ANOVA followed by the *post hoc* Holm-Sidak's multiple comparisons test.

Using aforementioned validated cell lines, we next evaluated the consequences of GMT cardiogenic transcription factor expression on CMC cytokine secretion. Conditioned medium (CM) sourced from GMT or GFP CMCs were analyzed using a Rat Proteome Profiler Array, allowing for the simultaneous antibody-mediated detection of 79 distinct soluble proteins on a single nitrocellulose membrane (Figure S2A). Densitometric quantification of resultant cytokine arrays revealed increased expression of a number of soluble factors in CM sourced from GMT CMCs relative to GFP controls (Figures S2B-S2D). These included cytokines implicated to function in pathways regulating matrix remodeling/collagen catabolism, cell proliferation/migration, as well as immune modulation (Figure S2E). Gene ontology (GO) analyses using the online DAVID bioinformatic resource database indicated similar predictions regarding their potential biological functions (Figure S3A-S3D). In fact, functional annotation clustering using GO terms highlights that many of these upregulated secreted proteins are constituents of the extracellular space (Figure S3A) and share common regional (e.g., extracellular matrix), as well as functional classifications, such as cell adhesion, migration, growth, and wound healing (Figure S3B-S3D). Based in part on these inferences, we postulated that GMT CMCs may exhibit augmented anti-fibrogenic paracrine signaling potency. To begin to address this notion, we sought to compare the ability of CM from GMT or GFP CMCs to impede TGFβ1-mediated cardiac fibroblast activation *in vitro*. Rat cardiac fibroblast activation efficiency was first validated via their stimulation with recombinant human TGFβ1 *in vitro* (Figures 2A and 2B). Relative to unstimulated fibroblast controls (incubated with bFGF), TGFβ1-treated cells exhibited morphometric features and marker expression prototypical to fibroblast activation (Figures 2A and 2B). This was characterized by a flattened cellular appearance, accompanied by the presence of distinguishable actin filament bundles (Figure 2A), and enhanced production of α-smooth muscle actin (α-SMA) and both fibrillar and non-fibrillar collagens (Col1A1 and Col19A1, respectively) (Figure 2B). Next, cardiac fibroblasts were concurrently incubated with TGFβ1 and CM from GMT or GFP CMCs to directly assess their competency to inhibit fibroblast activation *in vitro* (Figures 2C and 2D). Compared to TGFβ1 only controls, both CM from GMT and GFP CMCs yielded statistically significant reductions in the relative expression of Col1A1 (GFP CM 0.67 ± 0.04; GMT CM 0.52 ± 0.05) and Col19A1 (GFP CM 0.29 ± 0.05; GMT CM 0.33 ± 0.05, respectively) synthesis in TGFβ1-stimulated cardiac fibroblasts (Figures 2C-2F). Inhibition in collagen synthesis was not accompanied with modifications in SMA production, as neither GFP CM nor GMT CM produced a measurable change in α-SMA expression (Figure 2G). Although both GMT and GFP CM afforded equivalent reductions in Col19A1 synthesis (≈66-70%) in TGFβ1-activated cardiac fibroblasts relative to TGFβ1 only controls (Figure 2F), GMT CM more efficiently repressed Col1A1 synthesis by as much as 48%, compared to that of GFP CM, which resulted in only a 33% reduction (Figure 2E). Together, these results suggest that CMCs possess anti-fibrogenic paracrine signaling activity and, further, that such anti-fibrogenic properties are amplified with the expression of cardiogenic transcription factors.

### GMT CMCs afford a modest, yet quantifiable therapeutic advantage over naïve CMCs *in vivo*

**Figure 3:**
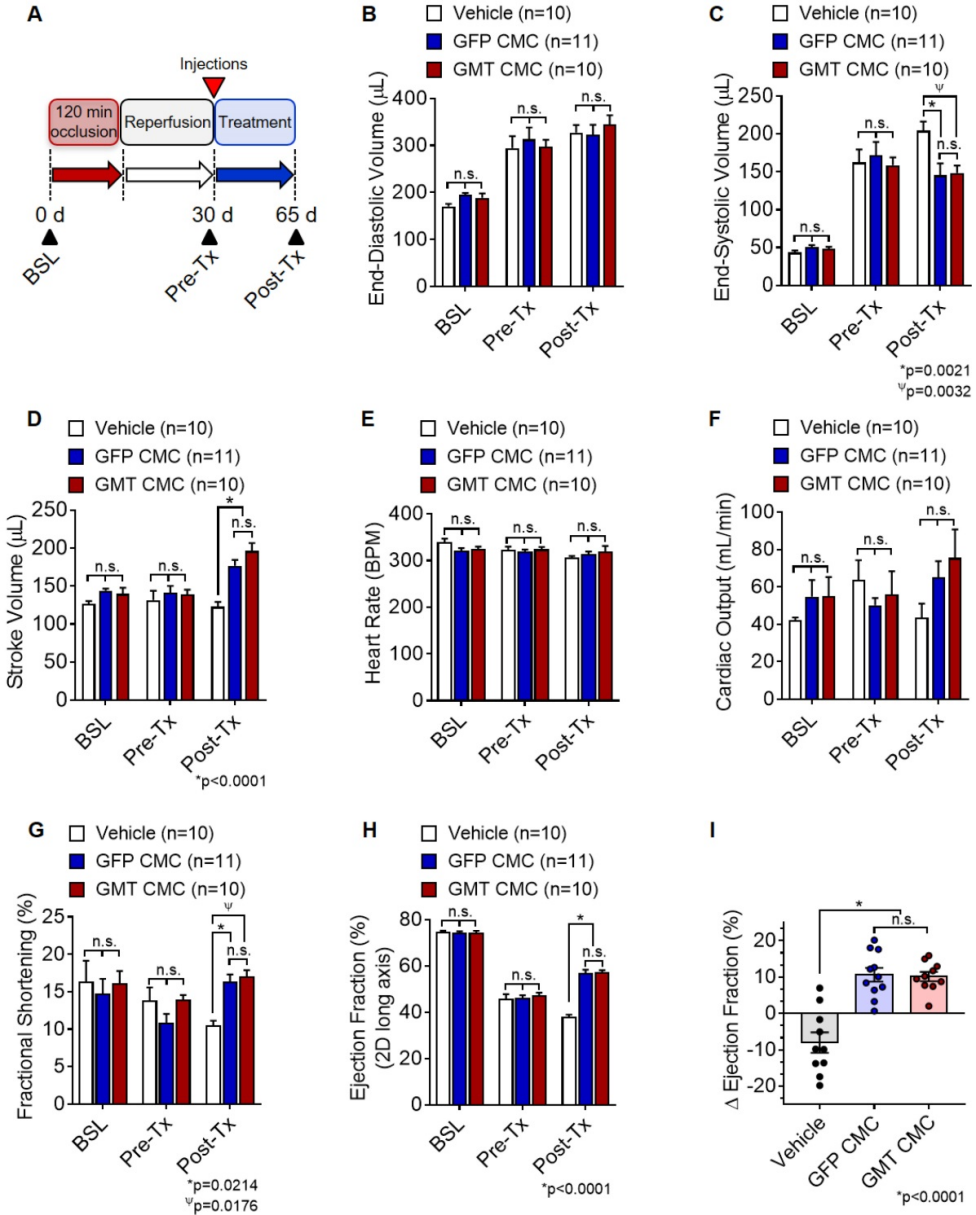
** Echocardiographic assessment of cardiac function.** (A) Schematic diagram detailing the timeline of ischemia-reperfusion procedures, serial echocardiogram collection (corresponding to baseline, pre-treatment [pre-Tx], and post-treatment [post-Tx]), and cell injections. At accompanying time points, ventricular volumes [(B) end-diastolic volume (µL) and (C) end-systolic volume (µL)] and functional parameters [(D) stroke volume (µL), (E) heart rate (BPM), (F) cardiac output (ml/min), (G) fractional shortening (%), and (H) ejection fraction (%)] were calculated for each group. The change in (I) ejection fraction (ΔEF) from pre-treatment to post-treatment is depicted in the accompanying dot plot. All graphs report mean ± SEM for treatment groups consisting of vehicle- (n=10), GFP CMC- (n=11), and GMT CMC- (n=10) injected animals. Data were analyzed via 1-way (panels (A-H)) or 2-way ANOVA (panel (I)) followed by the post hoc Holm-Sidak's multiple comparisons test.

Based on above findings, we predicted that GMT CMCs may be therapeutically superior to that of naïve CMCs (GFP CMCs). To test this hypothesis, F-344 rats were subjected to ischemia-reperfusion injury, randomized into treatment groups, and intramyocardially administered with CMCs (GMT CMC or GFP CMC) or PBS vehicle. Indices of cardiac function were assessed by serial echocardiograms on day 0 (baseline), day 30 (immediately prior to treatment; Pre-Tx), and day 65 (35 d after treatment; Post-Tx) (Figure 3A). As anticipated, echocardiographic determination of chamber dimensions confirmed the development of adverse left ventricular (LV) remodeling in vehicle-treated rats, as indicated via the progressive increase in both LV end-diastolic and end-systolic volumes (Figures 3B and 3C, respectively) compared to baseline. While vehicle-, GFP CMC-, and GMT CMC-treatment groups yielded no detectable difference in LV end-diastolic ventricular volumes 35 d after treatment (Figure. 3B), GFP CMC and GMT CMC-treated animals displayed a significant and comparable reduction in end-systolic ventricular volumes relative to that of vehicle controls (vehicle 204.3 ± 12.1 µL; GFP CMC 145.5 ± 15.7 µL; GMT CMC 148.6 ± 9.8 µL) (Figure 3C). Congruent with these observations, both GFP CMC and GMT CMC-treated rats exhibited improvements in stroke volume relative to vehicle-treated controls post treatment (Figure 3D); however, only a nominal advantage was observed with GMT CMCs compared to GFP CMCs (vehicle 122.8 ± 6.5 µL; GFP CMC 177.1 ± 7.6 µL; GMT CMC 196.4 ± 10.5 µL) (Figure 3D). Further, all cell-treated animals revealed sizeable, yet non-significant improvements in mean cardiac output relative to vehicle controls, with no calculable advantage in either CMC treatment group over the other (vehicle 43.7 ± 7.4 mL/min; GFP CMC 65.1 ± 8.7 mL/min; GMT CMC 75.5 ± 15.3 mL/min) (Figure 3F). Changes in mean cardiac output were not associated with alterations in heart rate, as all were shown to be similar among the different groups (Figure 3E). Additional indices of cardiac function, including fractional shortening (FS) and left ventricular ejection fraction (EF), were evaluated (Figures 3G-3I). Thirty-five days post treatment, both GFP- and GMT-treated CMCs produced comparable improvements in FS (vehicle 10.4 ± 0.7%; GFP CMC 16.3 ± 1.0%; GMT CMC 16.9 ± 1.0%) (Figure 3G) and left ventricular EF (vehicle 37.6 ± 1.3%; GFP CMC 56.6 ± 1.8%; GMT CMC 57.0 ± 1.1%) (Figure 3H) relative to vehicle controls. The change in EF (ΔEF) from pre- to post-treatment was also determined for each animal of each treatment group. Therein, both GFP and GMT CMC groups displayed robust improvements in ΔEF compared to vehicle (vehicle -8.0 ± 2.8%; GFP CMC +10.6 ± 1.9%; GMT CMC +10.2 ± 1.3%); however, there were no measurable differences in these indices between the two cell treatment groups (Figure 3I). Thus, echocardiographic evaluation suggests that ectopic expression of GMT does not yield a calculable supplemental benefit to the basal cardiac reparative capacity of CMCs *in vivo*.

**Figure 4:**
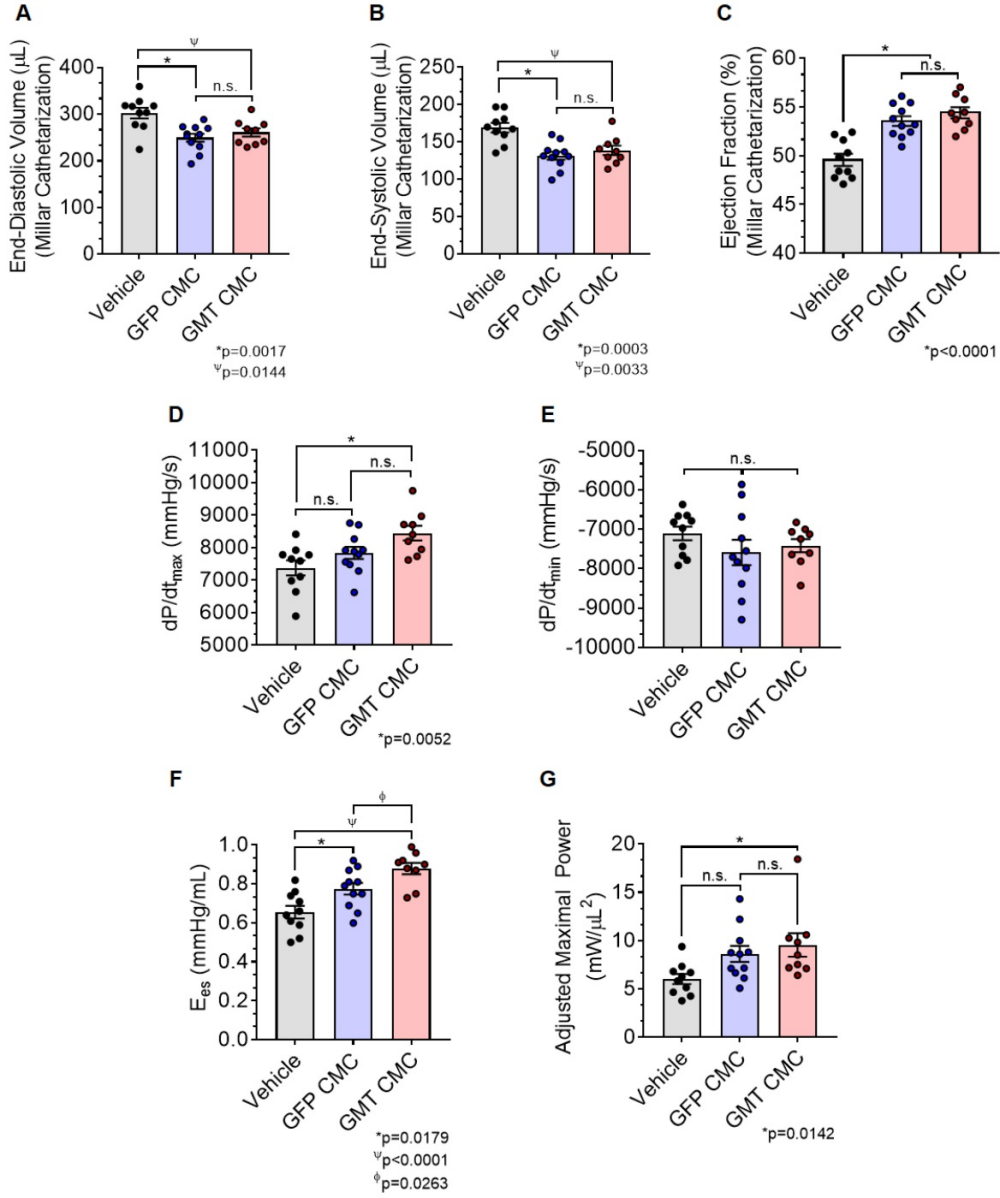
** Hemodynamic assessment of cardiac function.** Conductance catheters were used 35 days after CMC treatment to evaluate ventricular volumes [(A) end-diastolic volume (µL) and (B) end-systolic volume (µL)], (C) ejection fraction (%), (D) LV dP/dt_max_, (E) LV dP/dt_min_ (mmHg/sec), and load-independent indices of LV systolic function [(F) end-systolic elastance (mmHg/ml) and (G) pre-load adjusted maximal power (mWatts/µL^2^)]. All graphs report mean ± SEM for treatment groups consisting of vehicle- (n=10), GFP CMC- (n=11), and GMT CMC- (n=9) injected animals. All data were analyzed via one-way followed by the *post hoc* Holm-Sidak's multiple comparisons test.

As an additional measure, experimental groups were also subject to hemodynamic analyses thirty-five days after treatment (prior to euthanasia). Herein, the effects of CMC administration on load-dependent (LV ejection fraction, LV dP/dt_max_, and LV dP/dt_min_) and load-independent (end-systolic elastance (E_es_) and pre-load adjusted maximal power) indices of LV systolic function were assessed. Conductance catheterization revealed comparable, statistically significant reductions in both LV end-diastolic (vehicle 302.4 ± 11.6 µL; GFP CMC 249.7 ± 8.6 µL; GMT CMC 261.1 ± 8.9 µL) (Figure 4A) and end-systolic (vehicle 169.1 ± 6.4 µL; GFP CMC 131.5 ± 5.3 µL; GMT CMC 138.7 ± 6.3 µL) (Figure 4B) volumes in CMC-treated groups relative to vehicle controls. What is more, comparisons of ventricular function showed both GFP and GMT CMCs to produce measurable improvements in EF relative to vehicle-treated controls (Figure 4C) (vehicle 49.6 ± 0.6%; GFP CMC 53.6 ± 0.5%; GMT CMC 54.4 ± 0.6%). While both GFP and GMT CMCs exhibited statistical improvements in all of these evaluated endpoints, neither CMC treatment group possessed a discernible advantage over the other in terms of their ability to lessen ventricular dilatation or improve ejection fraction—conclusions which closely mirrored those derived from echocardiographic analyses. Surrogate measures of global LV contractility, such as maximal and minimal rate of developed LV pressure (dP/dt_max_ and dP/dt_min_, respectively), showed only GMT CMC-treated animals to possess a statistically significant improvement in dP/dt_max_ relative to vehicle controls (vehicle 7376 ± 231 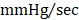; GFP CMC 7836 ± 185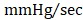; GMT CMC 8449 ± 224 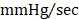) (Figure 4D); no differences in dP/dt_min_ were observed between the groups (Figure 4E). Observed combinatorial reductions in LV end-systolic volumes and increase dP/dt_max_ suggests that GMT CMCs may more efficiently enhance cardiac contractility than GFP CMCs *in vivo*. To this end, load-independent indices of LV contractility were comparatively measured between the groups. Said indices included both LV end-systolic elastance (E_es_) (Figure 4F) and preload-adjusted maximal power (Figure 4G). Congruent with this idea of more proficient enhancement of contractility, GMT CMCs on average produced a greater increase in E_es_ than that of GFP CMCs, relative to vehicle controls (vehicle 0.655 ± 0.033 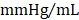; GFP CMC 0.776 ± 0.030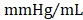; GMT CMC 0.879 ± 0.029 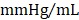) (Figure 4F). With regard to adjusted maximal power, only GMT CMC-treated animals displayed a statistically significant increase relative to vehicle controls—whereas only a nominal improvement was observed with GFP CMC-treated animals (vehicle 6.03 ± 0.53 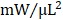; GFP CMC 8.63 ± 0.82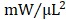; GMT CMC 9.55 ± 1.22 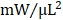) (Figure 4G). Taken together, two independent methods of functional assessment (echocardiography and hemodynamic measures) reproducibly demonstrate administered CMCs to improve LV systolic performance. Further, while conventional echocardiography was unable to detect a therapeutic advantage with GMT CMCs over that of GFP CMCs, hemodynamic analyses identified a modest, yet calculable supplemental benefit with GMT CMCs relative to naïve cells (GFP CMCs).

### GMT CMCs exhibit only modest enhancements in their capacity to impede myocardial collagen deposition *in vivo*

**Figure 5:**
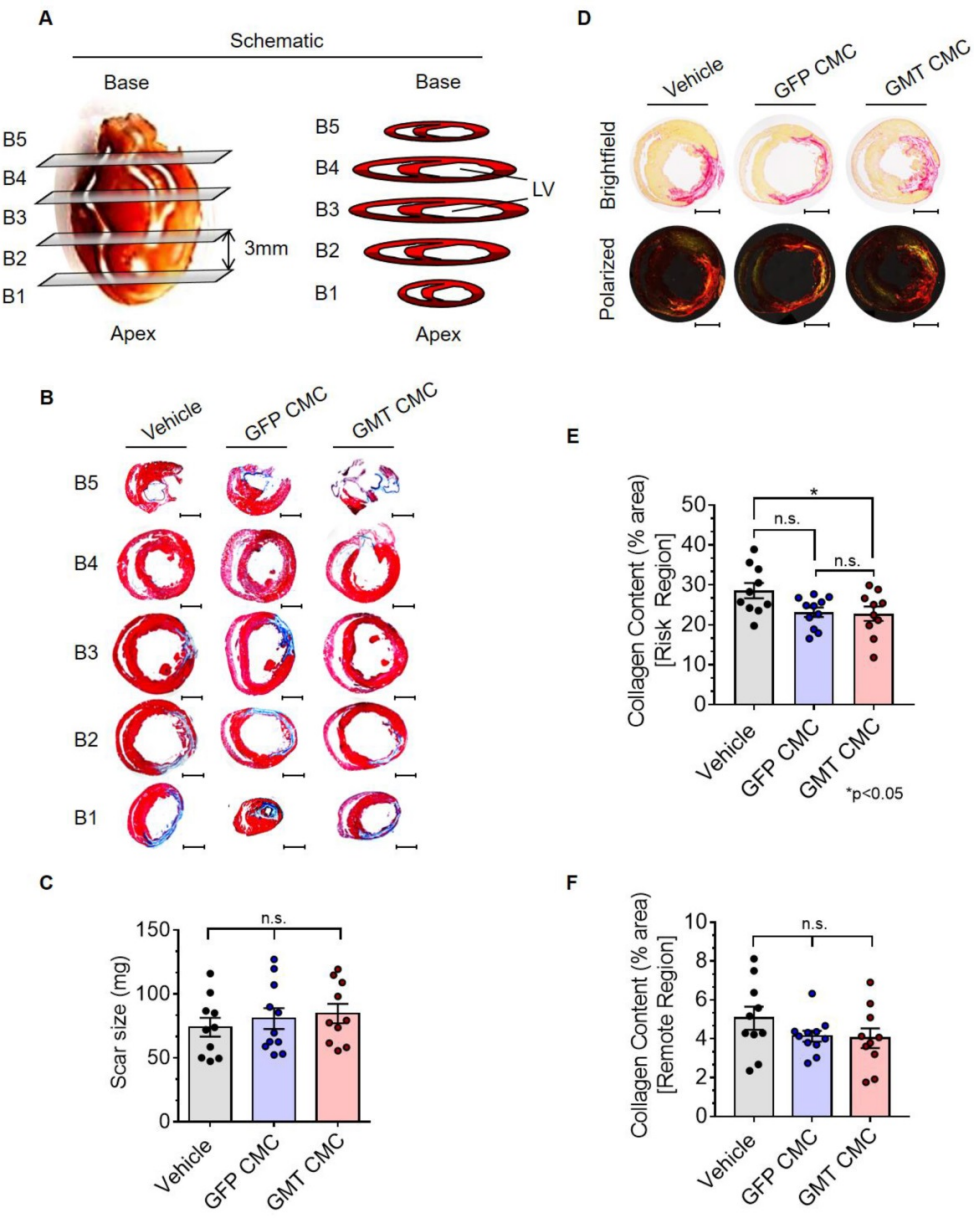
** Histopathological evaluation of infarct size and myocardial collagen content.** (A) Schematic diagram detailing cardiac dissection for histopathology. Each heart was dissected into 5 X 3 mm blocks (B1-B5) prior to histopathological analysis. Planimetric measurements of (B) Masson's trichrome-stained transverse myocardial tissue sections (B1-B5; 4 µm in depth) were used to enumerate scar size, reported in (C) mass (mg) in vehicle- (n=10), GFP CMC- (n=11), and GMT CMC- (n=10) treated rat hearts. Dot plots report the arithmetic mean ± SEM. Scale bars=3 mm. (D) Myocardial collagen content was digitally enumerated via polarized light microscopy of picrosirius red stained mid-papillary level myocardial sections (top row: brightfield; bottom row: polarized light). Scale bars=3 mm. Regional collagen content ((E) risk and (F) remote myocardial zones) was quantified in cardiac sections derived from vehicle- (n=10), GFP CMC- (n=11), and GMT CMC- (n=10) injected animals. Values are reported as regional collagen content (% area) ± SEM. Infarct size (Masson's trichrome) and myocardial collagen content (picrosirius red) data were analyzed via one-way ANOVA followed by the *post hoc* Holm-Sidak's and Tukey's multiple comparisons test, respectively.

Thirty-five days after administration, all groups were subjected to histological analyses to evaluate infarct size and myocardial collagen content. In Figure 5A, surgically resected hearts were transversally dissected into five consecutive blocks (B1-B5), from apex to base, in 3 mm increments. The scar size of each block was determined via computer-based planimetric measurements of Masson's trichrome-stained sections (Figure 5B) and summed to yield the total scar size (in mg) of each heart (Figure 5C). Histopathology results showed neither GFP nor GMT CMCs produced a measurable reduction in scar size relative to vehicle-treated controls (Figure 5C)—results which closely recapitulated our previous findings with human CMCs 20. Although cell therapy had no apparent effects on infarct size, alterations in myocardial collagen deposition were observed. In Figure 5D, regional collagen content was quantified by polarized light microscopy of picrosirius red stained, mid-ventricular level myocardial sections. Within the risk region (comprising the scar and border zone), GMT CMC-treated hearts displayed a statistically significant reduction in collagen content compared to vehicle, whereas only a nominal decrease was observed with GFP CMCs (collagen content [% area]: vehicle 28.6 ± 1.9%; GFP CMC 23.2 ± 1.2%; GMT CMC 22.8 ± 1.8%) (Figure 5E). This equated to a relative reduction in risk region collagen content of 22.6% versus 20.9% in GMT CMC- and GFP CMC-treated hearts, respectively, compared to vehicle controls (Figure 5E). Such anti-fibrogenic effects were less discernible in remote regions, whereas both GMT and GFP CMCs yielded similar, though non-significant, reductions in myocardial collagen content, relative to vehicle treatment groups (collagen content [% area]: vehicle 5.1 ± 0.6%; GFP CMC 4.1 ± 0.3%; GMT CMC 4.0 ± 0.5%) (Figure 5F). These results indicate that the intrinsic anti-fibrogenic activity of administered CMCs *in vivo* are modestly augmented by the ectopic expression of GMT core cardiogenic transcription factors.

## Discussion

Despite years of ongoing scientific investigation, the fundamental phenotypic properties governing the reparative potential of donor cells in cardiac cell therapy remain largely ambiguous, as are their rudimentary mechanism(s) of action. Previous investigations indicate that donor cell cardiomyogenic lineage commitment may be an important determinant of their therapeutic capacity 18, 20, 21, 37; however, whether this is a major element contributing to their salutary effects remains unanswered. Nevertheless, this idea has been the motivation behind a number of studies seeking to identify novel and effective strategies to directly reprogram mesenchyme towards a cardiomyogenic fate 20, 24-28—strategies which could hypothetically be leveraged to enhance the reparative aptitude of donor cells in cardiac cell therapy applications. Pursuing this course of investigation, we previously identified HDAC1 as a novel mediator of CMC cell fate decisions, wherein the targeted inhibition of HDAC1, via either shRNA-mediated depletion or entinostat (MS-275, SNDX-275) exposure, effectually promoted the activation of a core cardiogenic transcriptional program (e.g., *GATA4*, *MEF2C*, and *TBX5*) and cardiomyocyte-like differentiation of human CMCs *in vitro* 20, 25. In the latter study, this perceived enhancement in CMC cardiomyogenic differentiation with entinostat treatment was accompanied by their heightened aptitude to attenuate systolic dysfunction in a rat model of chronic ischemic cardiomyopathy 20. Interestingly, improvements in cardiac function were neither associated with enhancement in CMC retention nor the promotion of endogenous regenerative mechanisms (e.g., cardiomyogenesis, vasculogenesis, etc.), but with a greater reduction in myocardial collagen deposition 20. Consistent with this narrative, CMCs exposed to entinostat more robustly secreted cytokines associated with matrix remodeling and collagen catabolism 20—an observation which suggests, although indirectly, that their improved actions could be associated with lineage commitment-mediated adaptations in CMC anti-fibrogenic paracrine signaling. Indeed, pharmacologic HDAC inhibition afforded a tangible improvement in CMC cardiac reparative capacity, however, questions remained as to whether such improvements were the direct result of enhancement in CMC cardiomyogenic lineage commitment or simply off-target effects elicited by an otherwise non-specific epigenetic modifier. To this end, in the current study we utilized a more direct method (ectopic expression of GMT) to interrogate the consequences of augmented cardiomyogenic lineage commitment on CMC paracrine signaling dynamics and therapeutic capacity *in vivo*.

In the current study, we demonstrated ectopic expression of GMT cardiogenic transcription factors to effectually enhance CMC paracrine-mediated inhibition of collagen synthesis in *in vitro* activated cardiac fibroblasts. This was evidenced via conditioned medium-regulated blockade of the production of both fibrillar (Col1A1) and non-fibrillar (Col19A1) collagens *in vitro*. The extent by which CMC-derived conditioned medium reduced Col19A1 synthesis in activated fibroblasts was similar between GMT- and GFP-transduced CMCs (≈70% decrease in Col19A1); however, conditioned medium from GMT CMCs more efficiently impeded Col1A1 synthesis than that of GFP CMCs (48% vs. 33% decrease in Col1A1; GMT CMC vs. GFP CMC). This alone was an especially important observation as Col1A1 is a component of fibril-forming type I collagen that constitutes greater than 90% of cardiac fibrotic tissue 38. Further, fibrillar type I collagen itself is a chief structural component of the interstitium and its increased deposition and ultra-structure remodeling in the failing heart has long been known to adversely influence ventricular compliance/myocardial stiffness 39. Congruent with this observed enhancement in anti-fibrogenic paracrine signaling, cytokine array analyses of conditioned medium indicated that GMT CMCs more robustly secreted a number of soluble cytokines that function in molecular pathways involved in extracellular matrix remodeling, immune cell function, and proliferation and migration. Many of these included proteins directly involved in collagen degradation (e.g., MMP2 and MMP3) and cutaneous wound healing and tissue fibrosis (i.e., cellular communication network (CCN) family proteins: CCN1 (Cyr61), CCN3 (Nov), and CCN4 (Wisp-1)) 40, 41. Of the latter, CCN1 has been reported to induce fibroblast senescence and restrict fibrosis in cutaneous wound healing 40—phenomena which have recently been described in the neonatal mammalian heart 42. For instance, Feng and colleagues recently found that apical resection-induced CCN1 secretion from cardiomyocytes elicits fibroblast senescence and mitigates cardiac fibrosis in neonatal mice 42. Accordingly, these studies highlight CCN1, among those other upregulated CCN family members (CCN3 and CCN4), as potentially important contributors to the anti-fibrogenic paracrine signaling activities of GMT expressing CMCs—and perhaps even naïve CMCs. Although explicating the individual contributions of these factors to the anti-fibrogenic paracrine signaling potency of CMCs was beyond the scope of the current study, they constitute prime targets of interest in future investigations.

**Figure 6:**
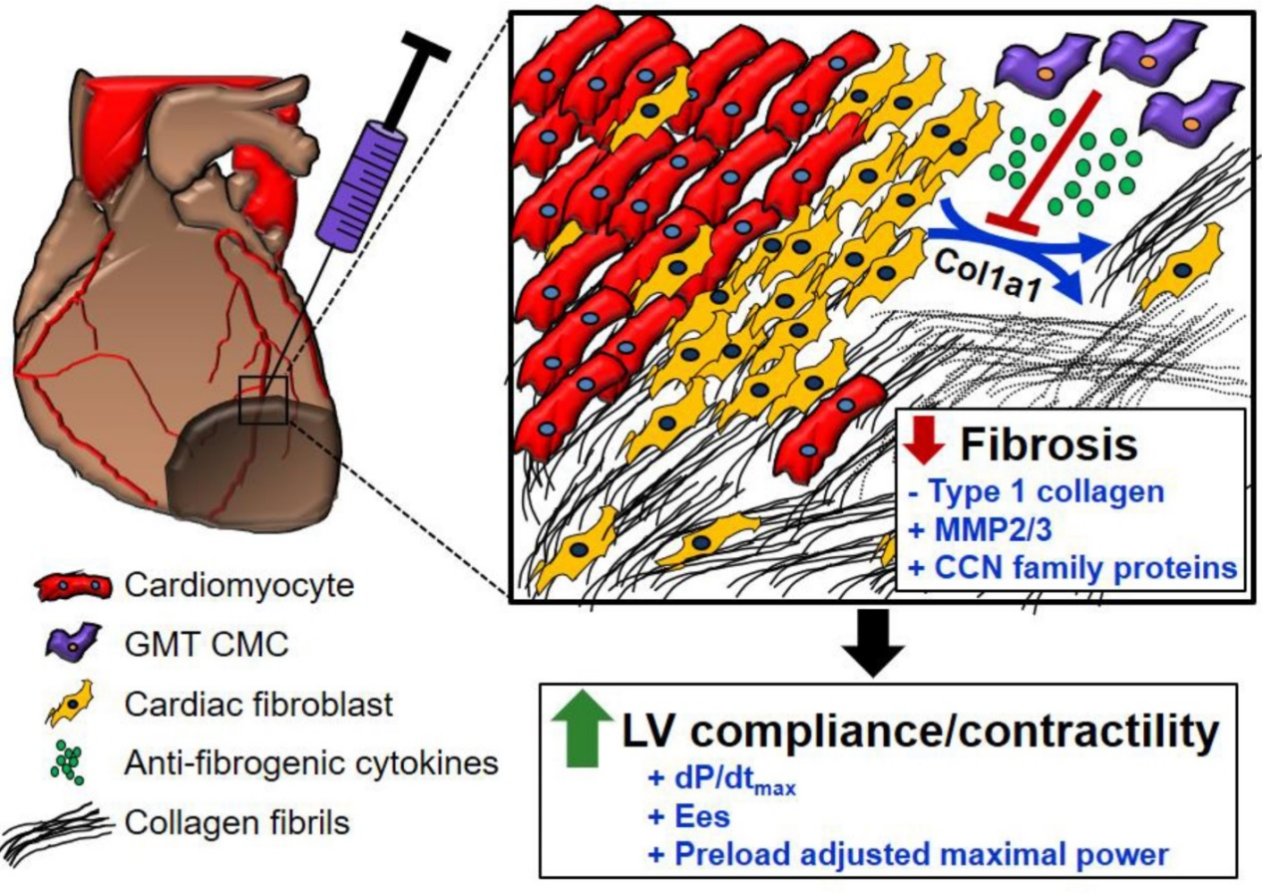
** Schematic detailing a proposed anti-fibrogenic mechanism of action.** GMT CMCs secrete a host of cardiotrophic factors, which include proteases (MMP2, MMP3) and matrix-associated proteins (CCN family proteins), that may correspondingly function to directly (through protease-mediated degradation of interstitial type I collagen), as well as indirectly (via paracrine-mediated inhibition of endogenous myofibroblast fibrillar Col1A1 production), retard myocardial collagen deposition. Such actions would support improved ventricular compliance and myocardial contractility in the absence of a change in surviving myocyte reserves.

In light of the above-mentioned observations, we hypothesized that ectopic expression of GMT cardiogenic factors would provide a supplemental benefit to the basal cardiac reparative capacity of CMCs *in vivo*. In the current study, echocardiographic analyses showed intramyocardially delivered GMT CMCs and GFP CMCs to yield comparable improvements in cardiac function 35 d after treatment—which most notably included significant reductions in end-systolic volume and marked improvements in stroke volume, fractional shortening, and ejection fraction. What is more, hemodynamic analyses largely reiterated many of these echocardiographic-based findings; therein, GMT and GFP CMCs produced equivalent reductions in ventricular volumes and similar enhancements in ejection fraction. While these two independent methods of functional assessment indicated that GMT CMCs possessed little, if any, therapeutic advantage over naïve CMCs (GFP CMCs), additional investigated physiologic parameters appeared to counter this assertion. For instance, indices of cardiac contractility, such as LV dP/dt_max_, LV end-systolic elastance (E_es_), and preload-adjusted maximal power exhibited statistically significant improvements in GMT CMC-treated animals relative to vehicle, whereas only nominal improvements in these measures were observed with GFP CMCs. Thus, while both GMT and GFP CMCs appeared to have markedly similar aptitude to improve LV ejection fraction, GMT expressing CMCs appeared to more ably increase myocardial contractility (i.e., E_es_)—a phenomenon which could be, in part, credited to their heightened capacity to impede myocardial fibrosis.^43^ Consistent with this notion and our previous results 20, histopathological evaluation of CMC-treated hearts indicated that improvements in ventricular function were neither associated with a decrease in infarct size nor an increase in viable myocardium, but with alterations in regional myocardial collagen content. Specifically, GMT CMCs exhibited statistically significant reductions in regional myocardial collagen deposition (risk region) relative to vehicle-treated controls, whereas only nominal reductions were observed with GFP CMCs. These anti-fibrogenic effects were less apparent within remote regions as both GMT and GFP CMCs produced comparable, yet non-significant reductions in myocardial collagen content. Collectively, these findings suggest that the intrinsic anti-fibrogenic activity and overall therapeutic capacity of administered CMCs are only modestly augmented by the ectopic expression of GMT core cardiogenic transcription factors—and therefore, the perceived benefits of GMT CMC therapy may not be physiologically significant. Nonetheless, these data support a proposed model in which the supplementary therapeutic benefits afforded by GMT cardiogenic factor expression are, in part, associated with heightened CMC anti-fibrogenic paracrine signaling potency (Figure 6). Such a mechanism is likely multifactorial, as CMCs appear to secret a myriad of cardiotrophic factors (i.e., MMP2/3 and CCN family proteins) that may respectively function to both directly (i.e., proteolytic degradation of interstitial type I collagen) and indirectly (i.e., inhibition of myofibroblast collagen production) impede myocardial fibrosis (Figure 6)—actions that may improve ventricular compliance and, in turn, enhance myocardial contractility.

A number of limitations were noted and should be considered when interpreting study findings. One such obvious limitation is the documented inefficiency of fibroblast cardiogenic reprogramming with ectopic expression of GMT cardiogenic factors alone 23, 36. For example, we and others have shown that cardiac-derived fibroblasts inefficiently acquire molecular phenotypes of mature cardiomyocytes when transduced with only GMT core cardiogenic transcription factors 23, 36. Hence, while the results of this study argue against donor cell lineage commitment as a major contributor to their therapeutic capacity, the failure of GMT transduction to efficiently promote the acquisition of a mature myogenic phenotype precludes such a proclamation. This in mind, complete cardiac reprogramming was not the intended goal of the current study, as we sought only to evaluate the contributions of the expression of these three HDACi-responsive cardiogenic factors 20, 25, namely GMT, on CMC paracrine signaling dynamics and therapeutic capacity. An additional limitation is the lack of consideration for the effect of different stoichiometric ratios of GMT factors within established CMC cell lines, as the relative expression/abundance of these factors have been shown to influence reprogramming efficiency 44. In fact, it was previously demonstrated that high protein levels of Mef2C and lower levels of Gata4 and Tbx5 significantly enhance the efficiency of fibroblast induced cardiomyocyte reprogramming 44. Notwithstanding, the tricistronic vector employed here was constructed with Gata4 in the leading position for reasons that we previously demonstrated Gata4, and not Mef2c and Tbx5, as the major potentiator of cardiogenic gene expression programs in cardiac-derived fibroblasts 23.

## Conclusions

In summary, we demonstrate that cardiogenic transcription factor expression exerts some influence on the anti-fibrogenic paracrine signaling potency and cardiac reparative capacity of donor cells. This suggests that lineage-specific adaptations in paracrine signaling dynamics may play, in part, a role in the underlying therapeutic actions of donor cells. These findings may be generalized to argue against the use of more developmentally primitive populations in cell therapy applications—and argue for the employment of cell types exhibiting a more committed cardiomyogenic phenotype.

## Supplementary Material

Supplementary figures and tables.

## Abbreviations

ACE: angiotensin-converting-enzyme
CCN: cellular communication network
CM: conditioned medium
CMC: cardiac mesenchymal stromal cells
DNMT: DNA methyltransferase
dP/dt_max_ and dP/dt_min_: maximal and minimal rate of developed LV pressure
E_es_: end-systolic elastance
EF: ejection fraction
GMT: GATA4, MEF2C, and TBX5 cardiogenic transcription factors
HDAC: histone deacetylase
HF: heart failure
LV: left ventricle
LVEDV: left ventricular end-diastolic volume
LVESV: left ventricular end-systolic volume
MMP: matrix metalloproteinase
MOI: multiplicity of infection
PV: pressure-volume
