# Supplementary material for: The Effect of Cardiogenic Factors on Cardiac Mesenchymal Cell Anti-Fibrogenic Paracrine Signaling and Therapeutic Performance
Source: Theranostics. 2020 Jan 1;10(4):1514–30. doi: 10.7150/thno.41000 (PMC6993223; doi:10.7150/thno.41000)
Supplement: Supplementary file 1 — Supplementary figures and tables. [file thnov10p1514s1.pdf]

Supplemental materials Section

Figure S1

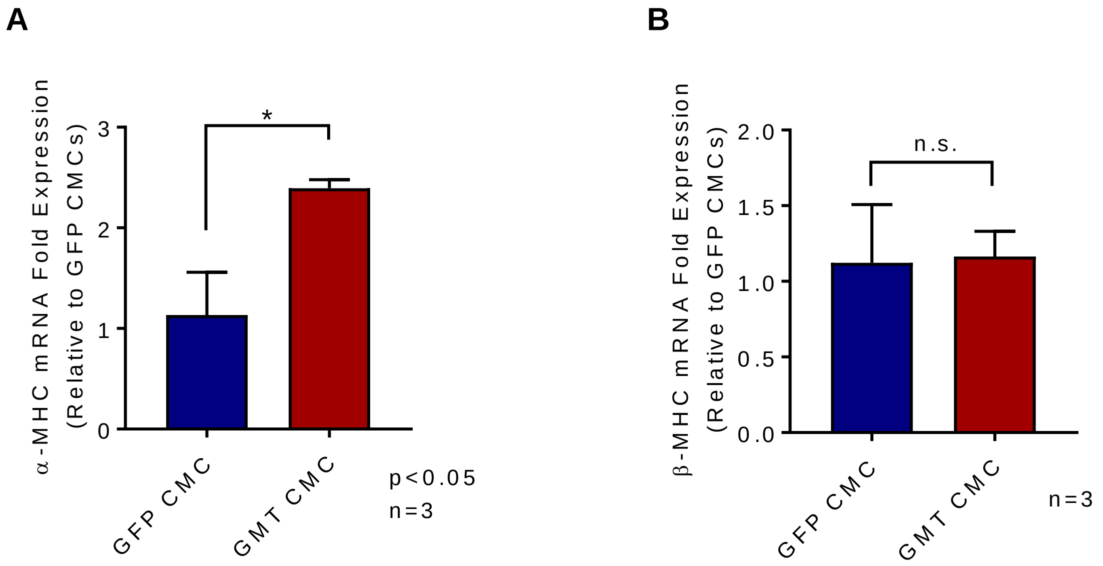

Figure S1. Effect of ectopic GMT expression on myosin cardiac markers.
GMT-transduced CMCs exhibit enhanced expression of (A) α-MHC, but not (B) β-MHC mRNA. Bar graphs denote the arithmetic mean of 3 independent biological replicates ± standard error. Formal tests for normality were performed using the Shapiro-Wilk normality test. P-values were calculated using a two-tailed, unpaired student's t-test.

Figure S2

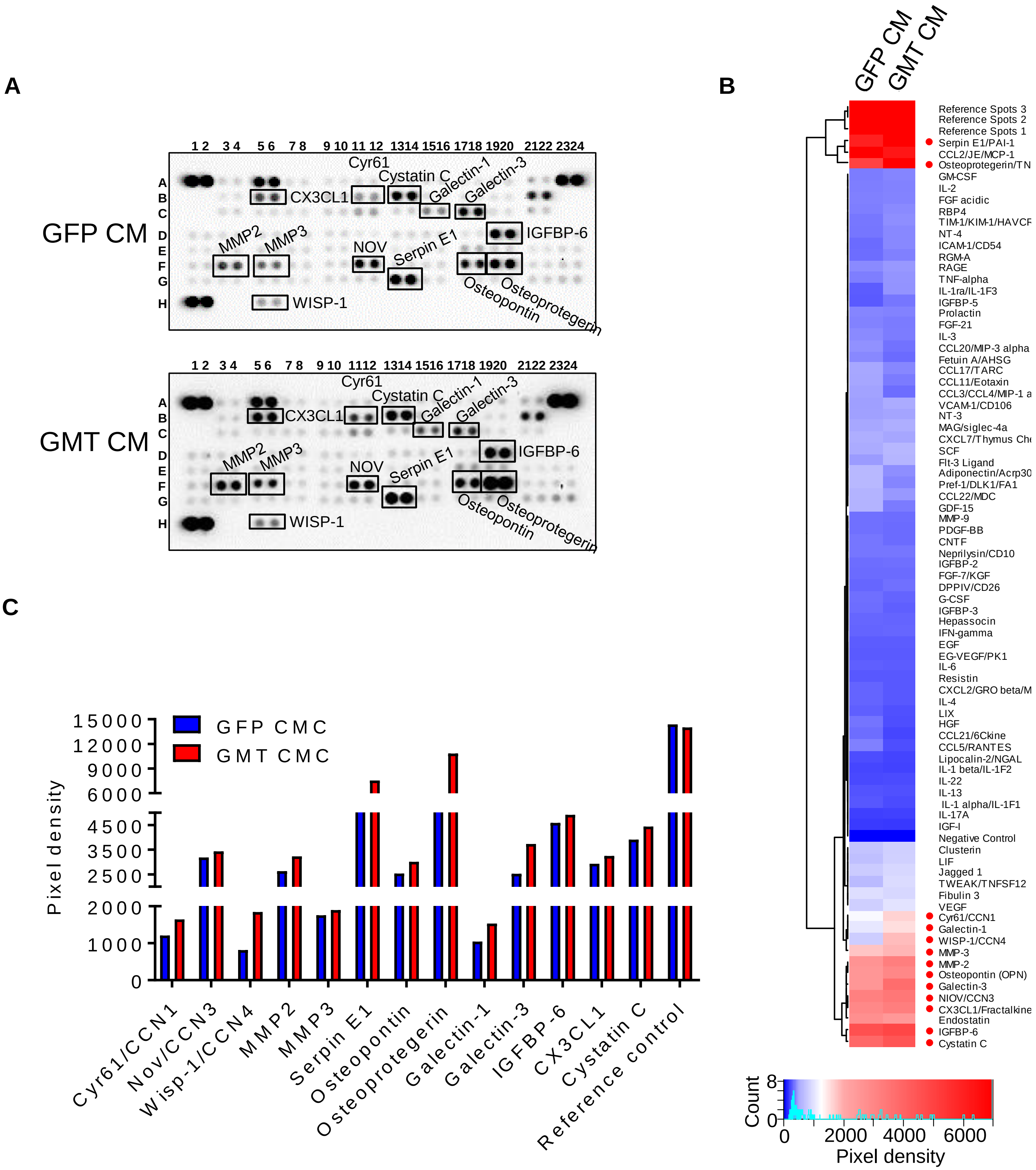
A
B
C
GFP CM
GMT CM
Cyr61
Cystatin C
Galectin-1
Galectin-3
CX3CL1
IGFBP-6
MMP2
MMP3
NOV
Serpin E1
Osteopontin
Osteoprotegerin
WISP-1
GFP CM
GMT CM
Reference Spots 3
Reference Spots 2
Reference Spots 1
Serpin E1/PAI-1
CCL2/JE/MCP-1
Osteoprotegerin/TN
GM-CSF
IL-2
FGF acidic
RBP4
TIM-1/KIM-1/HAVCF
NT-4
ICAM-1/CD54
RGM-A
RAGE
TNF-alpha
IL-1ra/IL-1F3
IGFBP-5
Prolactin
FGF-21
IL-3
CCL20/MIP-3 alpha
Fetuin A/AHSG
CCL17/TARC
CCL11/Eotaxin
CCL3/CCL4/MIP-1 a
VCAM-1/CD106
NT-3
MAG/siglec-4a
CXCL7/Thymus Che
SCF
Flt-3 Ligand
Adiponectin/Acrp30
Pref-1/DLK1/FA1
CCL22/MDC
GDF-15
MMP-9
PDGF-BB
CNTF
Neprilysin/CD10
IGFBP-2
FGF-7/KGF
DPPIV/CD26
G-CSF
IGFBP-3
Hepassocin
IFN-gamma
EGF
EG-VEGF/PK1
IL-6
Resistin
CXCL2/GRO beta/M
IL-4
LIX
HGF
CCL21/6Ckine
CCL5/RANTES
Lipocalin-2/NGAL
IL-1 beta/IL-1F2
IL-22
IL-13
IL-1 alpha/IL-1F1
IL-17A
IGF-I
Negative Control
Clusterin
LIF
Jagged 1
TWEAK/TNFSF12
Fibulin 3
VEGF
Cyr61/CCN1
Galectin-1
WISP-1/CCN4
MMP-3
MMP-2
Osteopontin (OPN)
Galectin-3
NIOV/CCN3
CX3CL1/Fractalkine
Endostatin
IGFBP-6
Cystatin C
Count
Pixel density
GFP CMC
GMT CMC
Pixel density
Cyr61/CCN1
Nov/CCN3
Wisp-1/CCN4
MMP2
MMP3
Serpin E1
Osteopontin
Osteoprotegerin
Galectin-1
Galectin-3
IGFBP-6
CX3CL1
Cystatin C
Reference control

Figure S2 (continued)

D

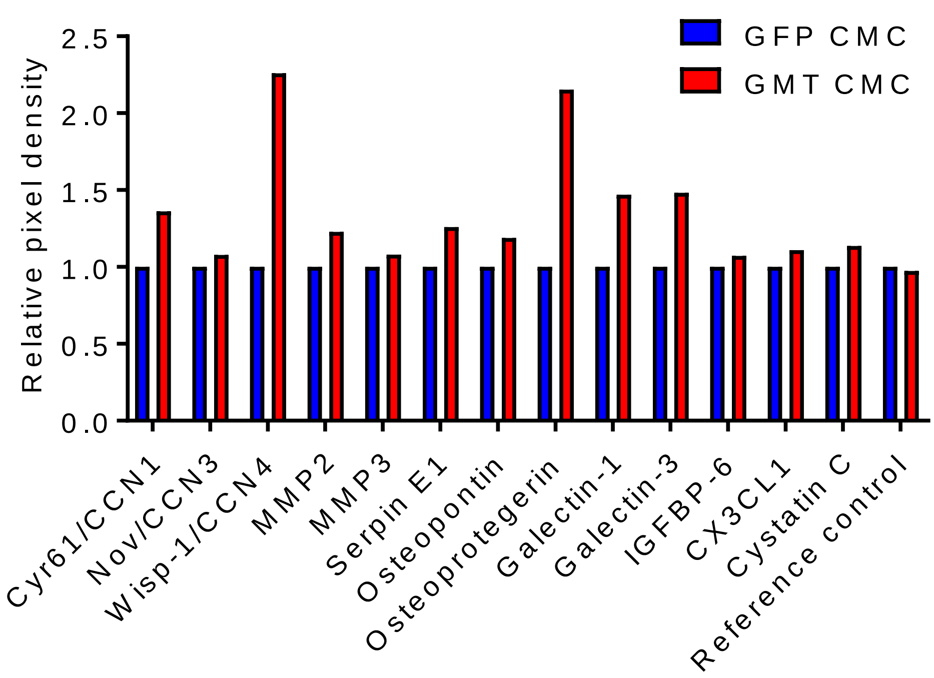

E

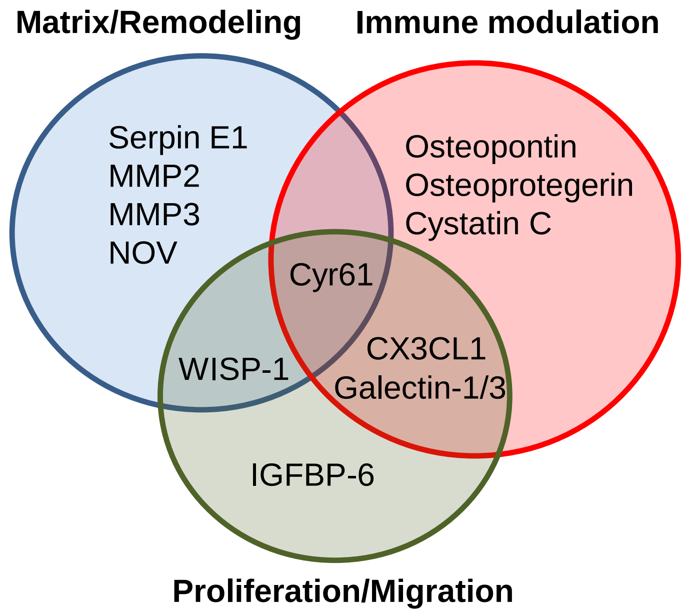

Figure S2. Ectopic GMT expression augments rat CMC cytokine secretion patterns
(A) Representative cytokine array. Conditioned medium (CM), derived from GFP- or GMT-transduced CMCs (n=2 biological replicates for each), was incubated with cytokine array membranes (Proteome Profiler Rat XL Cytokine Array Kit; R&D Systems, Minneapolis, MN) and detected using enhanced chemiluminescence. Black boxes highlight cytokines that are more abundant in CM from GMT CMCs compared to GFP CMCs. (B) Heatmap illustrating post hoc densitometric quantification of resultant cytokine array membranes. Total pixel density is reported. Red dots highlight upregulated cytokines in CM derived from GMT CMCs compared to GFP CMCs. In the figure image, low, medium, and high cytokine expression is indicated using blue, white, and red colors, respectively. Densitometric data graphically presented in bar graphs denoting (C) total pixel density and (D) relative pixel density (relative to GFP CM). (E) Venn diagram stratifying secreted cytokines, markedly upregulated in GMT-transduced CMCs, according to canonical biological processes in which they are known to be involved.

Figure S3

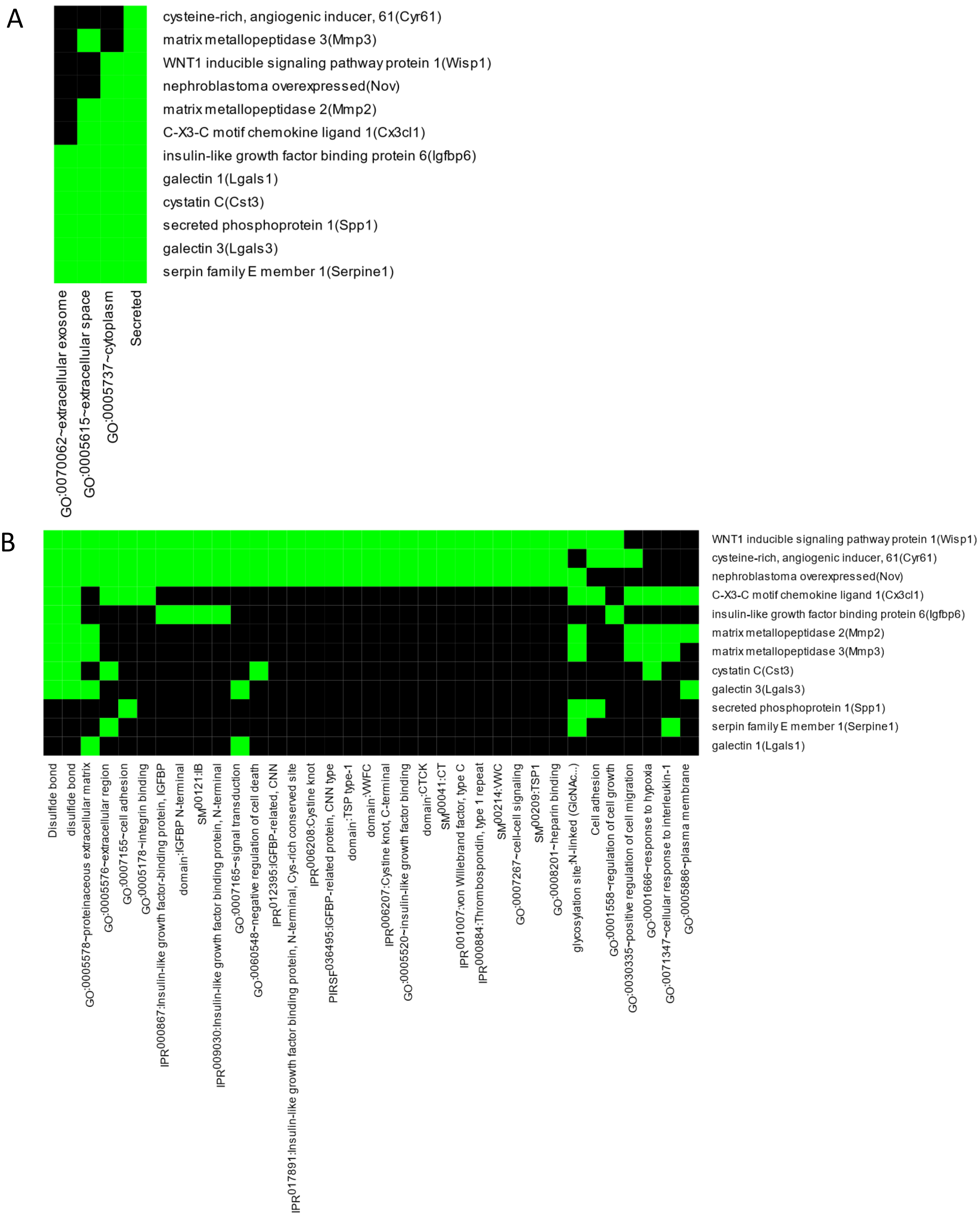
A
cysteine-rich, angiogenic inducer, 61(Cyr61)
matrix metallopeptidase 3(Mmp3)
WNT1 inducible signaling pathway protein 1(Wisp1)
nephroblastoma overexpressed(Nov)
matrix metallopeptidase 2(Mmp2)
C-X3-C motif chemokine ligand 1(Cx3cl1)
insulin-like growth factor binding protein 6(Igfbp6)
galectin 1(Lgals1)
cystatin C(Cst3)
secreted phosphoprotein 1(Spp1)
galectin 3(Lgals3)
serpin family E member 1(Serpine1)
GO:0070062~extracellular exosome
GO:0005615~extracellular space
GO:0005737~cytoplasm
Secreted
B
WNT1 inducible signaling pathway protein 1(Wisp1)
cysteine-rich, angiogenic inducer, 61(Cyr61)
nephroblastoma overexpressed(Nov)
C-X3-C motif chemokine ligand 1(Cx3cl1)
insulin-like growth factor binding protein 6(Igfbp6)
matrix metallopeptidase 2(Mmp2)
matrix metallopeptidase 3(Mmp3)
cystatin C(Cst3)
galectin 3(Lgals3)
secreted phosphoprotein 1(Spp1)
serpin family E member 1(Serpine1)
galectin 1(Lgals1)
Disulfide bond
disulfide bond
GO:0005578~proteinaceous extracellular matrix
GO:0005576~extracellular region
GO:0007155~cell adhesion
GO:0005178~integrin binding
IPR000867:Insulin-like growth factor-binding protein, IGFBP
domain:IGFBP N-terminal
SM00121:IB
IPR009030:Insulin-like growth factor binding protein, N-terminal
GO:0007165~signal transduction
GO:0060548~negative regulation of cell death
IPR012395:IGFBP-related, CNN
IPR017891:Insulin-like growth factor binding protein, N-terminal, Cys-rich conserved site
IPR006208:Cystine knot
PIRSF036495:IGFBP-related protein, CNN type
domain:TSP type-1
domain:VWFC
IPR006207:Cystine knot, C-terminal
GO:0005520~insulin-like growth factor binding
domain:CTCK
SM00041:CT
IPR001007:von Willebrand factor, type C
IPR000884:Thrombospondin, type 1 repeat
SM00214:VWC
GO:0007267~cell-cell signaling
SM00209:TSP1
GO:0008201~heparin binding
glycosylation site:N-linked (GlcNAc...)
Cell adhesion
GO:0001558~regulation of cell growth
GO:0030335~positive regulation of cell migration
GO:0001666~response to hypoxia
GO:0071347~cellular response to interleukin-1
GO:0005886~plasma membrane

Figure S3 (continued)

C

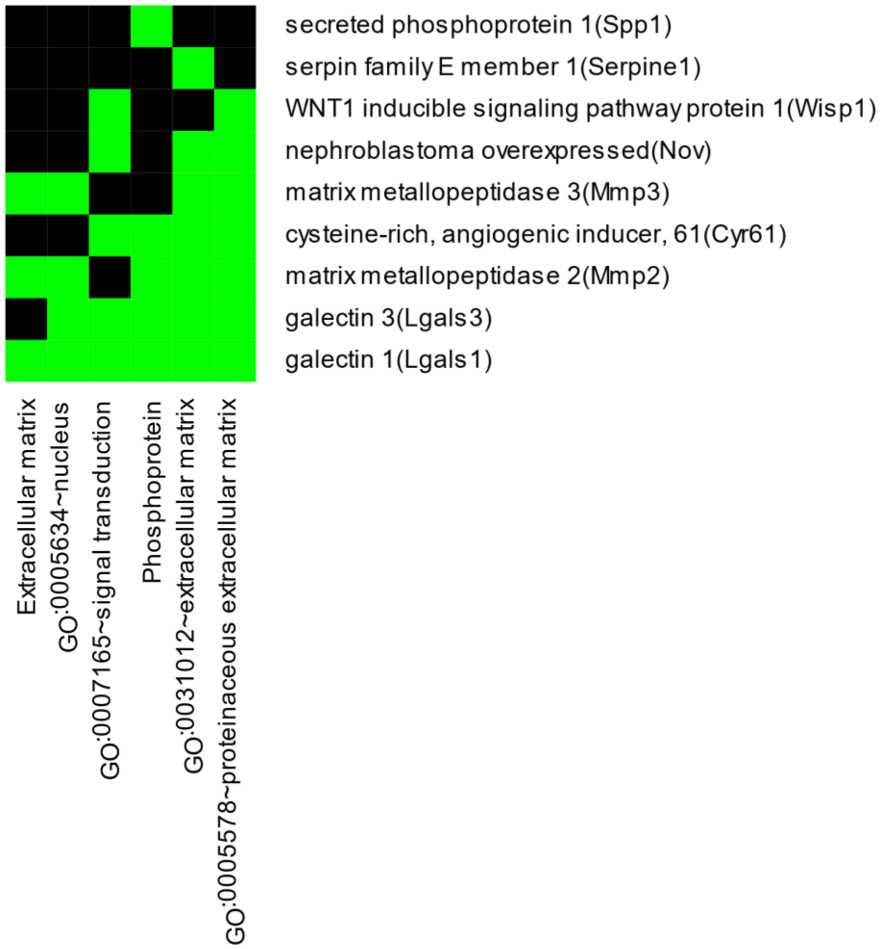

D

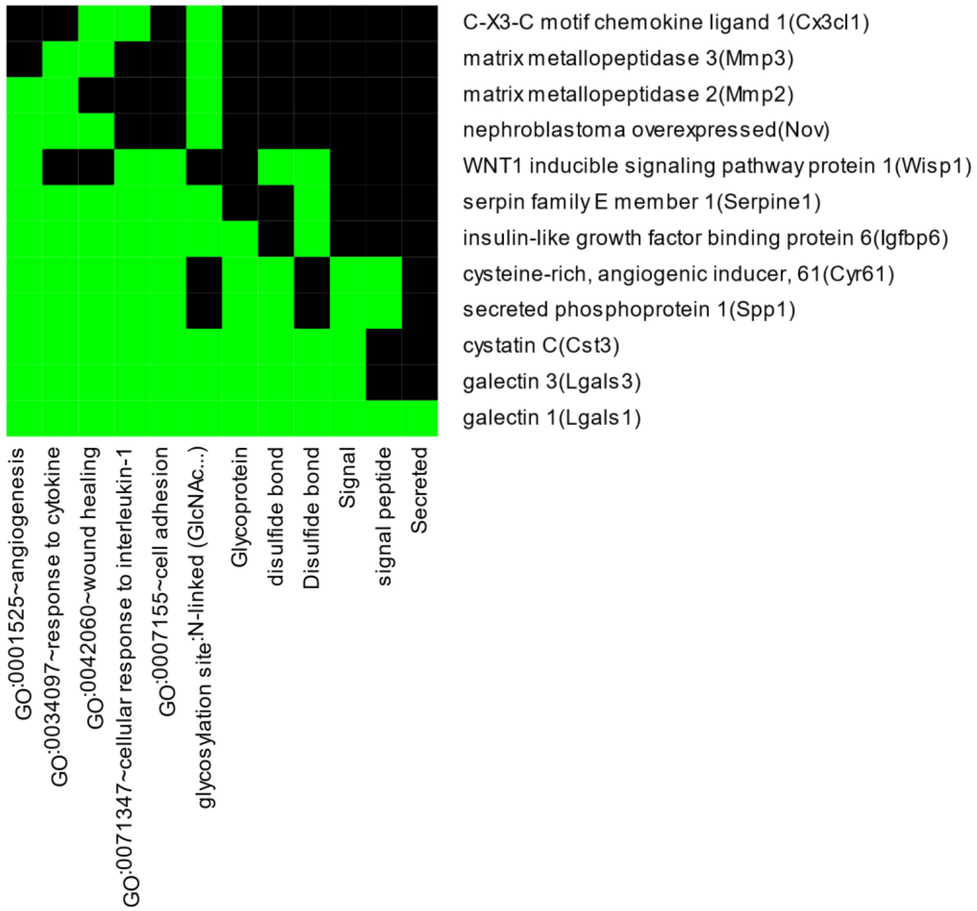

Figure S3. Gene ontology analyses of upregulated cytokines suggest GMT CMCs may exhibit augmented anti-fibrogenic paracrine signaling potency

(A-D) Functional annotation clustering using GO terms associated with those key soluble cytokines identified to be upregulated in condition medium sourced from GMT-transduced CMCs, relative to GFP-transduced controls. Green blocks indicate that a corresponding gene-term association has been positively reported. Black blocks indicate that a corresponding gene-term association has not been reported.

Table S1: Restriction cloning materials

Cloning primer (direction)	Restriction Enzyme	Primer Sequence (5´→3´)	Template	Amplicon (base pairs)
GATA4 (forward)	AgeI	TGC AAT ACC GGT ACC ATG TAC CAA AGC CTG G	pLenti6-3xFLAG-GATA4-V5[a]	1351 bp
GATA4 (reverse)	BsrGI	TAA CGT TGT ACA TCG CGG TGA TTA TGT CC		
MEF2C (forward)	XbaI	TGC AAT TCT AGA ACC ATG GGG AGA AAA AAG ATT CAG	pLenti6-3xFLAG-MEF2C-V5[a]	1427 bp
MEF2C (reverse)	BamHI	TAA CGT GGA TCC CTT TGT TGC CCA TCC TTC AGA AAG TCG		
TBX5 (forward)	BmtI	TGC AAT GCT AGC ATG GCC GAC GCA GAC G	pLenti6-3xFLAG-TBX5-V5[a]	1581 bp
TBX5 (reverse)	SalI	TAA CGT GTC GAC CTA GCT ATT GTC GCT CC		
Puro (forward)	PacI	TGC AAT TTA ATT AAA CTT TGG CCG CGG CTC G	pLK0.1-Puro[b]	1231 bp
Puro (reverse)	PacI	TAA CGT TTA ATT AAG AAC GTT ATT TGC GCT CCT TTC GGT CGG		

[a] Al-Maqtari T *et al*. (2017) Transcription factor-induced activation of cardiac gene expression in human c-kit+ cardiac progenitor cells. PLoS ONE 12(3): e0174242

[b] Sigma-Aldrich

Table S2: Antibodies employed

Antibody	Host	Conjugation	Supplier	Catalog #	Dilution	Exposure Time (minutes:seconds)	Predicted Molecular Weight (kDa)
Anti-GATA4 (D3A3M)	Rabbit mAb	Unconjugated	Cell Signaling Technology	36966	1:2000	1:30	55
Anti-MEF2C (D80C1)	Rabbit mAb	Unconjugated	Cell Signaling Technology	5030	1:1000	30:00	55
Anti-TBX5	Rabbit pAb	Unconjugated	Invitrogen	PA5-29845	1:2000	20:00	55
Anti-Col1A1	Rabbit pAb	Unconjugated	Cell Signaling Technology	84336	1:1000	4:00	250
Anti-Col19A1	Rabbit pAb	Unconjugated	ThermoFisher Scientific	PA5-23977	1:1000	15:00	130
Anti-α-Smooth Muscle Actin (D4K9N) XP	Rabbit mAb	Unconjugated	Cell Signaling Technology	19245	1:1000	0:20	55
Anti-β-Actin	Mouse mAb	Unconjugated	Invitrogen	AM4302	1:5000	0:25	55
Anti-rabbit IgG (secondary antibody)	Goat pAb	HRP-Linked	Cell Signaling Technology	7074	1:5000	N/A	N/A
Anti-mouse IgG (secondary antibody)	Horse pAb	HRP-Linked	Cell Signaling Technology	7076	1:5000	N/A	N/A
